# Monocyte signature as a predictor of chronic lung disease in the preterm infant

**DOI:** 10.3389/fimmu.2023.1112608

**Published:** 2023-04-05

**Authors:** Anita C. Windhorst, Motaharehsadat Heydarian, Maren Schwarz, Prajakta Oak, Kai Förster, Marion Frankenberger, Erika Gonzalez Rodriguez, Xin Zhang, Harald Ehrhardt, Christoph Hübener, Andreas W. Flemmer, Hamid Hossain, Tobias Stoeger, Christian Schulz, Anne Hilgendorff

**Affiliations:** ^1^ Institute of Medical Informatics, Justus-Liebig-University Giessen, Giessen, Germany; ^2^ Institute for Lung Health and Immunity and Comprehensive Pneumology Center, Helmholtz Zentrum München, German Center for Lung Research (DZL), Munich, Germany; ^3^ Department of Neonatology, Dr. von Hauner Childre's Hospital, University Hospital, Ludwig-Maximilian-University, Munich, Germany; ^4^ Center for Comprehensive Developmental Care (CDeCLMU) at the Social Pediatric Center, Dr. von Hauner Children`s Hospital, Ludwig Maximilian University (LMU) Hospital, Ludwig-Maximilian-University, Munich, Germany; ^5^ Division of Neonatology and Pediatric Intensive Care Medicine, University Medical Center Ulm, Ulm, Germany; ^6^ Department of General Pediatrics and Neonatology, Universities of Giessen and Marburg Lung Center (UGMLC), German Center for Lung Research (DZL), Justus-Liebig-University Giessen, Giessen, Germany; ^7^ Department of Gynecology and Obstetrics, Dr. von Hauner Children's Hospital, University Hospital, Ludwig-Maximilian-University, Munich, Germany; ^8^ Institute for Medical Microbiology, Justus-Liebig-University Giessen, Giessen, Germany; ^9^ German Center for Cardiovascular Research (DZHK), Partner Site Munich Heart Alliance, Munich, Germany; ^10^ Department of Medicine I, University Hospital, Ludwig Maximilian University, Munich, Germany

**Keywords:** bronchopulmonary dysplasia, monocytes, cytokines, preterm infants, prenatal injury, chronic lung disease

## Abstract

**Introduction:**

Inflammation is a key driver of morbidity in the vulnerable preterm infant exposed to pre- and postnatal hazards and significantly contributes to chronic lung disease, *i.e.* bronchopulmonary dysplasia (BPD). However, the early changes in innate immunity associated with BPD development are incompletely understood.

**Methods:**

In very immature preterm infants below 32 weeks gestational age (GA; n=30 infants), monocyte subtypes were identified by Flow Cytometry at birth and throughout the postnatal course including intracellular TNF expression upon LPS stimulation. Complementing these measurements, cytokine end growth factor expression profiles (Luminex^®^ xMAP^®^; n=110 infants) as well as gene expression profiles (CodeLink^TM^ Human I Bioarray; n=22) were characterized at birth.

**Results:**

The abundance of monocyte subtypes differed between preterm and term neonates at birth. Specifically, CD14^++^CD16^+^ (intermediate) monocytes demonstrated a dependency on PMA and elevated levels of nonclassical (CD14^+^CD16^++^) monocytes characterized preterm infants with developing BPD. Postnatally, lung injury was associated with an increase in intermediate monocytes, while high levels of nonclassical monocytes persisted. Both subtypes were revealed as the main source of intracellular TNF-α expression in the preterm infant. We identified a cytokine and growth factor expression profile in cord blood specimen of preterm infants with developing BPD that corresponded to the disease-dependent regulation of monocyte abundances. Multivariate modeling of protein profiles revealed FGF2, sIL-2 Rα, MCP-1, MIP1a, and TNF-α as predictors of BPD when considering GA. Transcriptome analysis demonstrated genes predicting BPD to be overrepresented in inflammatory pathways with increased disease severity characterized by the regulation of immune and defense response pathways and upstream regulator analysis confirmed TNF-α, interleukin (IL) -6, and interferon α as the highest activated cytokines in more severe disease. Whereas all BPD cases showed downstream activation of chemotaxis and activation of *inflammatory response pathways*, more severe cases were characterized by an additional activation of *reactive oxygen species (ROS) synthesis.*

**Discussion:**

In the present study, we identified the early postnatal presence of nonclassical (CD14^+^CD16^++^) and intermediate (CD14^++^CD16^+^) monocytes as a critical characteristic of BPD development including a specific response pattern of monocyte subtypes to lung injury. Pathophysiological insight was provided by the protein and transcriptome signature identified at birth, centered around monocyte and corresponding granulocyte activation and highlighting TNFα as a critical regulator in infants with developing BPD. The disease severity-dependent expression patterns could inform future diagnostic and treatment strategies targeting the monocytic cell and its progeny.

## Introduction

Organ injury provoked by sustained inflammation is especially detrimental in the developing organism. Perinatal inflammation is linked to increased mortality and long-term morbidity in preterm infants (1). One of the most prevalent morbidities bearing significant long-term consequences is the development of neonatal chronic lung disease (CLD), known as bronchopulmonary dysplasia (BPD) with different severities ranging from mild to moderate and severe disease (2). BPD affects more than 30% of all very preterm infants (3, 4) and determines the lung`s capacity to undergo structural and functional maturation as well as its potential to cope with second and third hit injury (5, 6).

The dysregulation of developmentally relevant growth factor signaling underlies the incomplete formation of the gas exchange area that is subsequently dominated by apoptotic changes and fibrotic remodeling. These processes are substantially driven by a sustained inflammatory response observed in the BPD lung (7). Despite several attempts to characterize these immune phenomena in the context of different pre- and postnatal risk factors driving BPD, comprehensive knowledge that informs diagnostic and therapeutic strategies at an early disease stage is missing. In the injured neonatal lung, both clinical and experimental studies have confirmed the presence of lung macrophages (MФ) and their respective signaling molecules (8, 9). These key players of innate immunity undergo a complex process of maturation and are in neonates characterized by shared and distinct functions when compared to adult MФ including lipopolysaccharide-induced activation, Fc receptor-dependent phagocytosis, and differences in polarization with interferon-gamma (IFNγ) or interleukin (IL)-10 (10, 11).

To understand early changes in the neonatal immune response that likely drive sustained inflammation related to BPD development on a cellular level and beyond, we characterized monocyte subtypes at birth and in the first week of life when undergoing postnatal treatments, delineated the corresponding cytokine and growth factor expression profile, and analyzed the immune network by transcriptome analysis in preterm infants with and without BPD.

## Material and methods

### Patient characteristics

Preterm infants <32 weeks gestational age (GA) with and without BPD were prospectively included in the study cohorts, following identical in- and exclusion (congenital malformations, metabolic disorders) criteria (12). Patient monitoring comprised an extensive set of clinical and laboratory variables from birth to discharge, including the variables displayed in **Table 1** (for definitions see table legend) (15). Specifically, BPD was diagnosed according to the NIH consensus statement (4) referring to oxygen requirement and/or ventilatory support at 36 weeks postmenstrual age (PMA) for disease severity. Incidences for BPD severity (no, mild, moderate and severe) are provided in each method section as well as in **Table 1** and **Supplemental Tables 1, 2**.

**Table 1:** Description of cohorts used for transcriptome, protein, and monocyte analysis.

	Monocyte analysis		Protein analysis		Transcriptome analysis	
	no BPD	BPD	no BPD	BPD	no BPD	BPD
**Number of patients**	15	15	55	45	13	9
**GA (weeks)**	30.14 (25.43-31.57)	25.86 (23.57-29.57)	30.6 (24.1-31.9)	26.6 (23.6 - 31.6)	30.3 (28.4-31.1)	24. 7 (24.3 - 30.3)
**Birth weight (g)**	1160 (750-1760)	590 (510-1510)	1310 (510-2240)	760 (315-1550)	1400 (900-1760)	830 (590-1390)
**Sex (female)**	8 (53%)	7 (47%)	27 (49%)	15 (33%)	6 (46%)	7 (78%)
**ANCS**	12 (80%)	12 (80%)	36 (67%)	27 (60%)	9 (69%)	7 (78%)
**AIS**	5 (33%)	11 (73%)	32 (64%)	30 (67%)	1 (8%)	5 (56%)
**RDS**	11 (79%)	15 (100%)	42 (42%)	43 (96%)	12 (92%)	9 (100%)
**EOI**	1 (9.1%)	5 (33%)	4 (7%)	16 (36%)	6 (46%)	9 (100%)
**Mechanical ventilation**	7 (47%)	15 (100%)	20 (36%)	39 (87%)	6 (46%)	9 (100%)
**Invasive ventilation (days)**	0 (0-9)	19 (3-42)	2 (1-53)	16 (1-40)	7 (2-9)	6 (1-44)
**CPAP/NIPPV (days)**	6.5 (2-50)	40.5 (34-59)	9 (1-48)	50 (0-119)	2 (1-13)	11 (3-45)
**Oxygen supplementation (days)**	0 (0-1)	63 (30-176)	5 (0-97)	63 (0-138)	4 (1-18)	46 (28-138)
**Surfactant therapy**	5 (33%)	13 (87%)	18 (33%)	37 (67%)	6 (46%)	9 (100%)

Data are given as median and range or frequencies (percent of total in group).

BPD was defined according to the NICHD/NHLBI/ORD workshop (4) based on the need for oxygen supplementation (>FiO2 0.21) for at least 28 days, followed by a final assessment at 36 weeks postmenstrual age (PMA) or at discharge, whichever came first in preterm infants born <32 weeks GA.

AIS, amniotic infection syndrome; ANCS, antenatal corticosteroids; CPAP, continuous positive airway pressure; EOI, presence of early postnatal systemic infections/early-onset infection [diagnosis: one or more clinical and laboratory signs of infection according to Sherman et al. (13)]; GA, gestational age; NIPPV, non-invasive positive pressure ventilation; RDS, respiratory distress syndrome [diagnosis and severity: assessment of anterior-posterior chest radiographs according to Couchard et al. (14)].

The study was approved by the local ethic committees at LMU Munich (ethic vote #195-07) and JLU Giessen (ethic vote #file-79**/**01).

For monocyte subtype analysis by FACS samples of n=30 infants were available for analysis. Protein analysis included samples from n=110 preterm infants, and samples from n=22 preterm infants were available for transcriptome analysis.

### Sample analysis

#### Flow cytometry (FACS)

In order to identify monocyte subtypes (16, 17) in volume limited (20-50µl) fresh, whole blood EDTA specimen of preterm infants with (n=15) and without (n=15) BPD, samples were incubated with CD45-APC (#IM2473), CD14(My4)-FITC (#6603511), CD16(3G8)-PE (#A07766) and HLA-DR-PC5 (#A07793) antibodies (Beckman Coulter, Germany) before adding blood volume equal amounts of counting beads (#7547053, Beckman Coulter, Germany). Data were analyzed on a four laser LSRII flow cytometer (Becton Dickinson, Germany). Monocyte subtypes were identified by gating on CD14/HLA-DR positive cells out of all CD45 positive leukocytes (**Supplemental Figure 6**). Further analysis for CD14 vs CD16 allowed us to define classical monocytes with high CD14 expression (CD14^++^CD16^-^), nonclassical monocytes with lower CD14 and high CD16 expression (CD14^+^CD16^++^), and intermediate monocytes with high CD14 and lower CD16 expression (CD14^++^CD16^+^) (18, 19). Data were analyzed by FlowJo^®^ version 10.8.1. Intracellular TNF staining was performed in whole blood samples (PerFix -nc, Beckman Coulter # B10826) according to the manufactures’ instructions. In brief, 50 µl of whole EDTA-blood and 10µg/ml Brefeldin A (Sigma #B-6542) were incubated with 100 ng/ml LPS (Lipopolysaccharide, Sigma #L6261) at 37°C for 4 hours or left without stimulus. Aliquots were stained after adding 2.5 µl Fixative Reagent 1 (15 min, RT) using 150 µl Permeabilizing Reagent 2 and monoclonal antibodies CD14-PC5 (Beckmann Coulter #A07765), CD16(3G8)-FITC (Becton Dickinson #555406) and TNF-PE (Beckman Coulter #IM3279) or TNF-PE + rTNF in a 10-fold molar excess as an isotype control (incubation for 20 min at RT). After adding 800 µl 1:10 diluted Final Reagent B, cells were analyzed using a LSR II flow cytometer. Monocyte subtypes were identified while gating on CD14-PE versus CD16-FITC. Corresponding iTNF expression was calculated while subtracting the mean fluorescence intensity of TNF-PE minus TNF-PE/rTNF for the respective monocyte subtypes and then calculating the delta mean fluorescence intensity after stimulation with LPS for the three analyzed monocyte subpopulations.

#### Protein analysis

For protein analysis, venous umbilical cord blood (CB) of 110 preterm infants (no BPD n=55, BPD (mild/moderate/severe) n=45) was centrifuged (1,000 rpm, 5 min) and stored (-20°C). All samples were analyzed using the 21-plex premixed human cytokine milliplex panel (HPANLXM 2, Luminex^®^ xMAP^®^, Luminex, TX, US) according to the manufacturer’s instructions.

#### Transcriptome analysis

For transcriptome analysis, venous umbilical CB specimens of 22 preterm infants (no BPD n=13, BPD n=9), were stabilized using the PAXgene Blood RNA System (PreAnalytiX, Germany) before RNA extraction (PreAnalytiX) and quantification (NanoDrop Technologies, US). From a total of 61 array images for analysis, spot signals of CodeLink^TM^ Human I Bioarrays (GE Healthcare/Amersham Biosciences) were quantified according to the manufacturer’s instructions (12) (CodeLink System Software (Batch Submission (V2.2.27), Expression Analysis (V2.2.25), GE Healthcare, Germany). Two to three technical replicates were prepared per sample. Microarrays were background corrected by subtracting and intra-slide normalized using Median normalization as recommended by the manufacturer. The 9945 transcripts were filtered for missingness (threshold >= 50% missing data per group), low expression (threshold >= 50% of values expressed below the detection threshold as defined by the manufacturer), and outliers per transcript probe and group over all microarrays (values deviating from the group median by more than 3). The remaining missing values were imputed by Bayesian Principal Component Analysis imputation in R (‘pcaMethods’ (20),). Subsequently, data were inter-slide normalized using Quantile normalization. The average expression of technical replicates was used for analysis. Transcriptome data were archived in Gene Expression Omnibus (GEO, Accession number: GSE225881).

### Data analysis

Data analysis was conducted in R (Version 4.1.1) (21).

*Monocyte subtypes* were analyzed using a linear mixed-effects model (R-Package *nlme* (22) that was fitted by maximizing restricted log-likelihood in order to model log-transformed monocyte levels dependent on clinical conditions, i.e., BPD, preterm or term birth, PMA, status of invasive positive pressure ventilation (timepoints: birth, before IPPV, start of IPPV, during and 2 weeks after IPPV). To analyze the development of monocyte levels over time, random effects were added to the statistical model.

*Protein expression* was analyzed by the Wilcoxon test; orthogonal partial least squares discriminant analysis (OPLS ***-*** DA) (22) for multivariate data sets using the Bioconductor packages ‘ropls’ (version 1.24.0), gestational age was included in the analysis. Variable influences on projection (VIP) were calculated to explain intergroup variation, i.e., BPD and no BPD. Orthogonal VIPs were calculated to facilitate the interpretation and detection of latent variables in the protein assay explaining intragroup variation. VIPS greater than 1 are considered most relevant, VIPS smaller than 0.5 are considered irrelevant (23).

*Transcriptome expression* analysis included prediction [PAM; Bioconductor packages ‘pamr’ (version 1.56.1)] (24, 25) and differential expression analysis (linear modeling, limma open source software, Bioconductor release 3.14) (26). Predictive analysis for microarray data used a multivariate approach to differentiate between BPD and BPD severity grades (no BPD n=13, mild BPD n=6, moderate or severe BPD n=3), while differential analysis for microarray data focused on differences in single transcripts in a univariate approach. The significance level was adjusted for multiple testing using the False Discovery Rate (FDR) (27). Cut-off FDR for differential expression was set at FDR < 0.05 and a minimum absolute fold change of 2 between at least two groups.

## Results

### Characteristic monocyte signature in infants with BPD at birth

**Figure 1:**
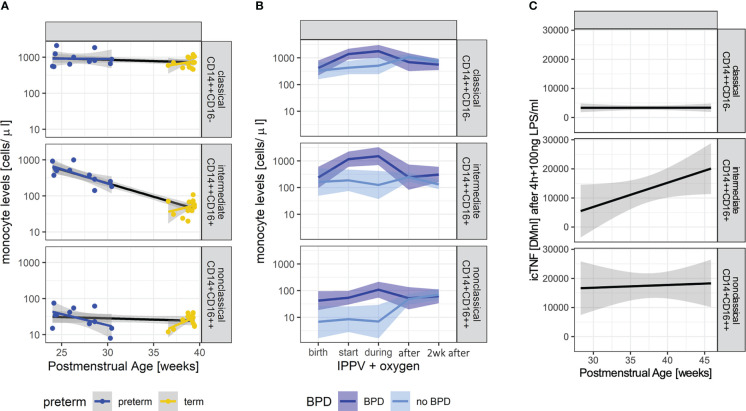
Characteristic monocyte subtypes at birth in infants developing BPD. **(A)**: Monocyte levels (upper panel: classical (CD14^++^CD16^-^), middle panel: intermediate (CD14^++^CD16^+^), lower panel: nonclassical CD14^+^CD16^++^) of preterm (n=11, blue) and term (n=14, yellow) infants at the day of birth (+72h if not done immediately). Overall trend dependent on PMA indicated by black lines. Group comparisons: abundancy preterm vs. term 0.695, p-value=0.067; development with PMA (slope): -0.017, p-value= 0.003. **(B)**: Monocyte levels (upper panel: classical (CD14^++^CD16^-^), middle panel: intermediate (CD14^++^CD16^+^), lower panel: nonclassical CD14^+^CD16^++^) in preterm infants with (n=15, dark blue) and without BPD (n=15, light blue) at birth up until 2 weeks after the start of invasive positive pressure ventilation (IPPV) and oxygen therapy. Linear mixed effect models take PMA into account. Levels of nonclassical monocytes are elevated at birth (pairwise comparison, p-value=0.004); nonclassical (BPD vs. no BPD p-value < 0.001), intermediate (BPD vs. no BPD p-value=0.011) and classical (BPD vs. no BPD p-value=0.004) differ during IPPV. **(C)**: Intracellular TNFα (icTNF) expression in response to LPS stimulation (4 hours after) in monocytes of preterm infants (n=9) (upper panel: classical (CD14^++^CD16^-^), middle panel: intermediate (CD14^++^CD16^+^), lower panel: nonclassical CD14^+^CD16^++^). Increases in icTNF are statistically significant in intermediate (p-value = 0.005) and nonclassical monocytes (p-value = 0.033). Levels of icTNF are displayed as delta mean intensities (DMnI).

In order to identify early postnatal immune cell characteristics that indicate or drive morbidity development, we characterized monocyte subtypes in volume-limited CB and follow-up samples from n=30 preterm infants with and without BPD. Supporting a GA specific pattern of innate immune capacities, each monocyte subpopulation showed individual trajectories (p<0.05, two-way Analysis of Variance). Whereas levels of intermediates and nonclassical monocytes at birth decreased with increasing PMA towards stable expression levels exhibited by term newborns, classical (CD14^++^CD16^-^) monocytes showed PMA independent abundances at birth (**Figure 1A**). The abundance of CD14^++^CD16^+^ (intermediate) monocyte subtypes differed between preterm and term neonates at birth (p-value=0.067) and demonstrated a dependency on PMA (p-value=0.003) (**Figure 1A**). When considering disease, our analysis revealed elevated levels of nonclassical CD14^+^CD16^++^ monocytes in preterm infants with developing BPD at birth (p-value=0.004) (**Figure 1B** and **Table 2**). In the postnatal course, lung injury, *i.e.*, exposure to oxygen and invasive positive pressure ventilation (IPPV) was associated with an increase of CD14^++^CD16^+^ (intermediate) monocytes (p-value < 0.001), while high levels of CD14^+^CD16^++^ monocytes persisted (**Figure 1B** and **Table 2**). A peak of all monocyte subtypes can be observed when preterm infants with developing BPD are exposed to oxygen and IPPV in contrast to infants without the disease (**Figure 1B** and **Table 2**). With regard to cellular function, intermediate CD14^++^CD16^+^ and nonclassical CD14^+^CD16^++^ monocytes from preterm infants were revealed as the main source of intracellular TNF-α expression upon LPS stimulation (**Figure 1C**). The capacity of CD14^++^CD16^+^ for TNF-α expression upon stimulation increased with PMA (**Figure 1C**).

**Table 2:** Comparison of monocyte levels with before, during, and after start of invasive positive pressure ventilation (IPPV).

timepoint	classical CD14++CD16-	intermediates CD14++CD16+	nonclassical CD14+CD16++
birth	-0.04 +/-0.21, p-value=0.8662	0.01 +/-0.32, p-value=0.9796	**1.05 +/-0.32,** **p-value=0.0036**
start IPPV	0 +/-0.2, p-value=0.9972	0.12 +/-0.31, p-value=0.7141	-0.04 +/-0.3, p-value=0.8914
during IPPV	**0.7 +/-0.21,** **p-value=0.0044**	**0.93 +/-0.32,** **p-value=0.011**	**1.47 +/-0.32,** **p-value=0.0003**
after IPPV	-0.17 +/-0.13, p-value=0.2236	0.04 +/-0.19, p-value=0.8501	0.05 +/-0.19, p-value=0.7896
2 wks after IPPV	-0.11 +/-0.11, p-value=0.3388	*0.28 +/-0.15*, *p-value=0.086*	0.02 +/-0.14, p-value=0.8863

Repeated measurement analysis of variance for each monocyte subpopulation (classical, intermediate, nonclassical) with postmenstrual age as covariate. P-values and estimates for differences in log(cells) as well as standard deviation of comparison of BPD (n=15) vs no BPD (n=15) are displayed for each timepoint. Bold values show significance at a 95%-significance level; p-values < 0.05.

Next, we identified a cytokine and growth factor expression profile by multiplexed protein analysis in CB EDTA plasma from n=110 infants that corresponded to the disease dependent regulation of monocyte abundances in preterm infants with developing BPD. Protein profiling demonstrated the upregulation of IL-6, IL-8, MCP-1, MIP1a, IL-1Rα, sIL-2 Rα, EGF, and FGF2 levels in preterm infants with BPD when compared to infants without the disease. Multivariate modeling revealed FGF2, sIL-2 Rα, MCP-1, MIP1a, and TNF-α as predictors of BPD when considering GA as a strong influencing factor (VIP). Further, TNF-α, IL1b, IL6, IL8, and IL-1Rα were identified as top mediators for protein abundance (**Table 3**, oVIPs). In line with these findings, transcriptome analysis in CB EDTA samples of 22 very preterm infants identified a cluster of 28 transcripts differentiating between BPD and no BPD cases (**Supplementary Figure 1** and **Supplementary Table 1**) with a cluster of 71 transcripts differentiating between disease severities (**Supplementary Figure 2** and **Supplementary Table 2**). Genes predicting BPD are significantly overrepresented in *inflammatory* and *Galanin/GMAP prepropeptide* pathways (**Supplementary Table 3**) and increased disease severity is characterized by the regulation of immune and defense response pathways (**Supplementary Table 4**) including activated *chemotaxis of cells*, increased biological functions associated with *apoptosis*, *accumulation of leukocytes*, and decreased *phagocytosis by immune cells* (**Supplementary Table 5**). Differential gene expression analysis revealed 238 differentially expressed genes (DEG) out of 7,529 transcripts (FDR <0.05, and |FC|>2) when comparing cases with and without BPD (**Supplementary Table 6**). Upstream regulator analysis indicated TNF-α, interleukin (IL)-2, -6, -10, and interferons as the highest activated cytokines in moderate/severe BPD patients (**Supplementary Table 7**).

**Table 3:** Comparison of protein abundance at birth.

Protein	n	BPD (n=45)	no BPD (n=55)	p-value Wilcoxon-Test	oVIP	VIP
**GA**	100	26.6 (25.1-27.9)	30.6 (29.2-31.0)	<0.001	0.03	3.53
**EGF**	100	107.5 (86.9-372.3)	87.8 (66.7-118.9)	0.008	1.00	0.50
**FGF2**	98	135.1 (70.4-231.2)	96.6 (54.0-141.4)	0.032	0.88	1.23
**GCSF**	99	96.5 (57.5-392.8)	113.4 (62.3-209.6)	0.673	1.12	0.26
**GMCSF**	100	3.2 (3.2-6.2)	3.2 (3.2-5.6)	0.827	1.07	0.40
**IFNg**	100	3.2 (3.2-3.2)	3.2 (3.2-3.2)	0.904	1.04	0.02
**IL-10**	99	7.0 (3.2-13.7)	6.4 (3.2-11.7)	0.547	1.09	0.35
**IL-12p40**	100	14.8 (3.2-41.4)	13.4 (3.2-46.3)	0.603	1.01	0.56
**IL-1a**	100	18.1 (4.9-36.1)	12.2 (3.4-30.5)	0.191	0.98	0.49
**IL-1b**	100	3.2 (3.2-3.2)	3.2 (3.2-3.2)	0.193	1.29	0.20
**IL1ra**	100	55.6 (25.1-215.3)	22.4 (5.1-86.2)	0.023	1.24	0.25
**IL–4**	100	3.2 (3.2-3.2)	3.2 (3.2-3.2)	0.515	0.02	0.45
**IL-6**	100	9.6 (3.8-30.4)	3.3 (3.2-25.5)	0.031	1.26	0.65
**IL-8**	100	53.4 (31.8-107.0)	24.6 (11.7-53.1)	<0.001	1.26	0.69
**IP10**	100	200.1 (151.6-435.6)	190.1 (121.8-321.2)	0.369	0.93	0.47
**MCP1**	100	951.3 (545.5-1798.4)	596.3 (384.8-983.2)	0.005	1.12	1.15
**MCP3**	100	3.2 (3.2-3.2)	3.2 (3.2-3.8)	0.096	0.10	0.24
**MIP1a**	98	18.8 (3.2-28.4)	14.5 (3.2-22.7)	0.051	0.91	1.13
**MIP1b**	100	53.9 (38.1-94.2)	53.0 (37.4-66.4)	0.509	1.07	0.35
**sIL2Rα**	100	206.6 (100.4-402.7)	115.7 (47.9-274.9)	0.022	0.63	1.21
**TNF-α**	100	15.7 (10.8-19.7)	12.1 (9.8-17.2)	0.117	1.32	1.09
**VEGF**	96	119.4 (84.2-246.9)	136.2 (93.8-186.8)	0.887	0.98	0.10

Univariate analysis for protein values obtained by Wilcoxon tests and multivariate analysis using orthogonal partial least squares discriminant analysis (OPLSDA). Multivariate analysis considered also gestational age (GA). Median and upper and lower quartile are presented for continuous variables, n is the number of non-missing values. VIP, Variable influence on projection; oVIP, orthogonal Variable influence on projection from the orthogonal partial least squares discriminant analysis modeling BPD. GA, gestational age; protein names according to 21-plex premixed human cytokine milliplex panel.

### Mechanistic insight into BPD immune signature by transcriptome analysis at birth

**Figure 2:**
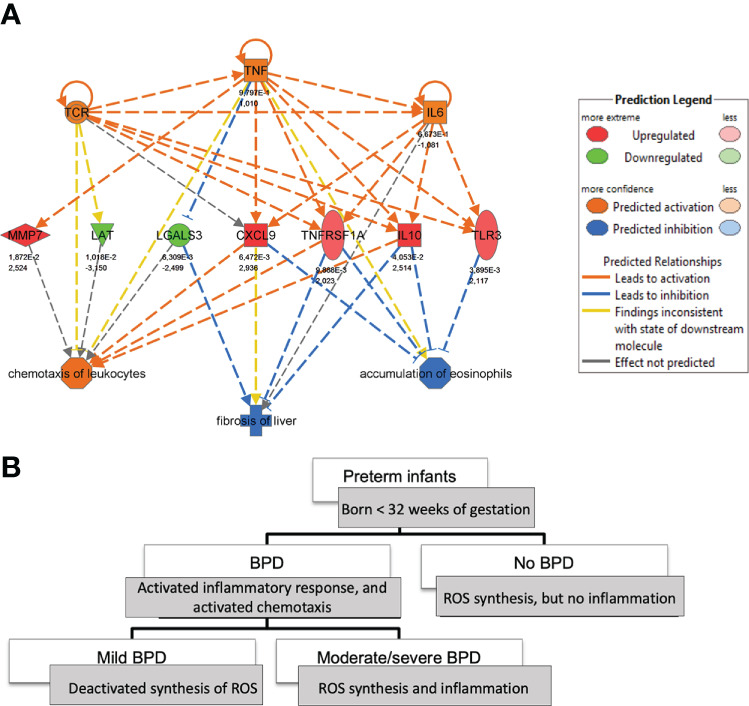
Tracking of monocyte-related cytokine expression in BPD at birth. **(A)**: Regulator-effects-network highlighting the role of TNFα in mild BPD (n=6) in comparison to no BPD (n=13) at birth. The network is derived from Ingenuity Pathway analysis of the transcripts differentiating between BPD severity grades (up- and downstream). **(B)**: The comparison of downstream effects of transcripts, able to predict BPD severity grades at birth, demonstrates involvement of inflammatory response, chemotaxis, and synthesis of Reactive Oxygen species (ROS) depending on BPD severity.

Regulator effect networks consisting of upstream cytokines and downstream biological function demonstrated the activation of IL-6, TNF-α, and TNFRSF1A (tumor necrosis factor receptor superfamily, member 1a) together with the regulation of *CXCL9, LGALS3, MMP7, TLR3, IL-10*, as well as *TCR* and *LAT* in infants with BPD, indicating immune cell activation, cell-matrix interaction and remodeling at the same time. Prediction analysis revealed the activation of TNF-α as a possible regulator for the differentially regulated genes *CXCL9, IL10, LGALS3, MMP7, TLR3, TNFRSF1A* (**Figure 2A**), and also *BID, CAT, CDH13, TF, ZFP36* (**Supplementary Figure 3**), with functional analysis confirming the activation of *leukocyte chemotaxis*, increased neutrophil number, increased *apoptosis and necrosis* and the deactivation of the *accumulation of eosinophils* together with the regulation of *TNF-α homeostasis* (*ZFP36*, *TNFRSF1A*). Increased disease severity was characterized by *reactive oxygen species (ROS) synthesis*, whereas all BPD cases independent of disease severity showed downstream activation of chemotaxis and activation of inflammatory response pathways (**Figure 2B** and **Supplemental Figure 4**). The transcriptome signature that was dominated by immune system activation in upstream analysis, indicated the upregulation of IL-2, TNF-α IL-6, IL-10, Interferon (IF) alpha/beta, and IFNG in mild BPD, next to the involvement of interferons from the alpha group, IL27, EBI3 in moderate/severe BPD. When comparing mild and moderate/severe BPD, differences included the activation of IL-5 (upregulation in moderate/severe BPD), and WNT1 (**Supplementary Table 8**).

## Discussion

There is a paucity of data providing insight into the immune cell characteristics that indicate or drive pulmonary morbidity in preterm infants early after birth.

Significantly extending clinical and experimental studies that associated interleukin and cytokine expression levels with pre-and postnatal injury and adverse long-term development in preterm infants (28, 29), we were able to identify the early postnatal presence of nonclassical and intermediate monocytes as a critical component in BPD development including their response pattern to lung injury. This picture was supported and pathophysiologically deepened by the protein and transcriptome signature identified, centered around monocyte and corresponding granulocyte activation.

In our study, BPD infants were characterized by elevated levels of CD14^+^CD16^++^ (nonclassical) monocytes and demonstrated a peak of all monocyte subtypes studied upon lung injury, especially revealing an increase in intermediate monocytes. Identified as the main source of stimulated intracellular TNF-α expression in preterm neonates, the early postnatal predominance of nonclassical and intermediate monocytes in infants with lung injury that develop BPD was closely reflected by the immune response identified in protein and transcriptome profiling.

Results from multiplexed proteomics demonstrated the regulation of IL-6, IL-8, MCP-1, MIP1a, IL-1Rα, sIL-2 Rα, EGF, and FGF2 in BPD infants at birth. The signature is related to *monocyte activation*, *proliferation*, *chemotaxis*, and *recruitment* (30), and statistical modeling revealed the potential of these cytokines and growth factors to both predict BPD as well as mediate protein abundance in these infants under strong consideration of TNF-α. These results aligned with transcriptome analysis, where upstream regulator analysis identified TNF-α, interleukin (IL)-2, -6, -10, and interferons as the highest activated cytokines in BPD patients with moderate or severe disease.

The identification of TNF-α as an upstream regulator of gene expression and protein abundance in BPD in concert with the overall cytokine and growth factor signature described, links back to the predominance of nonclassical and intermediate monocytes observed in BPD infants, as they represent a main source of TNF-α production as shown by us and others (31, 32) Regarding functional relevance for BPD development, TNF-α, and interferon-gamma signaling are not only known to drive monocyte to macrophage polarization enhancing extravasation of these cells and accumulation of monocyte-derived alveolar macrophages, but they as well exhibit significant profibrotic potential in lung disease (33).

The image of monocyte and granulocyte activation portrayed by the protein and transcriptome analysis is further illustrated by the network that we identified to characterize preterm infants with BPD as early as birth. Here, regulator effect networks demonstrated the activation of IL-6, TNF-α, and TNFRSF1A side by side with the regulation of genes involved in immune cell activation, cell-matrix interaction, and remodeling (CXCL9, LGALS3, MMP7, TLR3, IL-10, TCR, LAT) in infants with BPD. Differential regulation of genes involved in leukocyte chemotaxis and accumulation of eosinophils (CAT, CDH13, IL-10, LGALS3, TNFRSF1A, BID, TF, ZFP36) were again predicted to be regulated by TNF-α. The underlying network of immune signals identified by functional transcriptome analysis further enabled us to associate increased disease severity with the activation of genes involved in leukocyte chemotaxis and apoptosis side-by-side with a decrease in phagocytosis, thereby closely reflecting findings of studies on neonatal MФ function in BPD (10). Whereas all BPD cases, independent of disease severity, showed reactive oxygen species (ROS) synthesis, the main differences between mild and more severe BPD cases included the engagement of adaptive immune responses (IL-5) and developmental pathways (WNT1), in line with previous studies (34, 35).

Our results are supported by studies in preterm infants that demonstrated a predictive signature for inflammatory lung disease in preterm infants by transcriptional profiling of lung macrophages, highlighting IL-6, TNF, and CCL3 to be regulated in BPD, as well as studies that engaged protein profiling and revealed a comparable immune signature (36). Supported by a characteristic transcriptome and protein signature, the predominance of the nonclassical and intermediate monocyte subtypes identified in our study likely reflects critical processes for BPD development, including their impact on proliferative state, telomere length, cellular ROS levels, and mitochondrial membrane potential as critical functions in aging and cellular senescence (32, 37). The revelation of distinct developmental pathways from circulating monocytes to lung macrophages highlights the potential of specific monocyte subtypes to differentially contribute to populations of alveolar, interstitial, and pulmonary intravascular macrophages (38). While nonclassical blood monocytes give rise to intravascular macrophages in the lung, classical monocytes have been associated with interstitial and alveolar macrophages (38, 39). Intermediary CD14+ monocytes expressing macrophage markers exist reflecting stages of monocyte-macrophage transition (40). The role of monocyte-derived macrophages in tissue development (38), including their impact on growth factor signaling as well as their relation to a pro-fibrotic phenotype (33) further underlines their potential as critical regulators of BPD development (41, 42).

The disease-characteristic immune response aligns with the maturational effects observed in the monocyte subtypes studied, as intermediate and nonclassical monocyte levels both demonstrated an association with PMA and specifically intermediate (CD14^++^CD16^+^) monocytes were increased in premature infants at birth. The capacity of these cells to express TNF-α upon stimulation, however, increased with maturation.

Closing the knowledge gap or early drivers in chronic lung disease development, we successfully identified distinct signatures of monocyte subtypes in volume-limited CB specimen and follow-up samples in preterm infants with BPD, complemented by comprehensive protein and transcriptome profiling.

The identified immune response in preterm infants with developing BPD at birth holds potential for the design of diagnostic and therapeutic strategies but warrants future studies engaging large patient collectives to delineate the differential impact of prenatal complications as well as genetic background on these disease characteristic profiles.

## Data availability statement

The data presented in the study are deposited in the GEO repository (https://www.ncbi.nlm.nih.gov/geo), accession number GSE225881.

## Ethics statement

This study was confirmed by the local ethic committees at LMU Munich (ethic vote #195-07) and JLU Giessen (ethic vote #file-79/01), following the same in- and exclusion (congenital malformations, metabolic disorders) criteria. The patients/participants provided their written informed consent to participate in this study.

## Author contributions

AH, HH, TS, and CS designed the study. PO, CH, KF, MF, AF, EG, and XZ acquired the data. AH, AW, MH, PO, HE, MS, MF, and HH analyzed and interpreted the data. AH, AW, MH, TS, and CS drafted the manuscript for important intellectual content. All authors contributed to the article and approved the submitted version.
