# Corrigendum: Monocyte signature as a predictor of chronic lung disease in the preterm infant

**DOI:** 10.3389/fimmu.2023.1209992

**Published:** 2023-04-28

**Authors:** Anita C. Windhorst, Motaharehsadat Heydarian, Maren Schwarz, Prajakta Oak, Kai Förster, Marion Frankenberger, Erika Gonzalez Rodriguez, Xin Zhang, Harald Ehrhardt, Christoph Hübener, Andreas W. Flemmer, Hamid Hossain, Tobias Stoeger, Christian Schulz, Anne Hilgendorff

**Affiliations:** ^1^ Institute of Medical Informatics, Justus-Liebig-University Giessen, Giessen, Germany; ^2^ Institute for Lung Health and Immunity and Comprehensive Pneumology Center, Helmholtz Zentrum München, German Center for Lung Research (DZL), Munich, Germany; ^3^ Department of Neonatology, Dr. von Hauner Childre’s Hospital, University Hospital, Ludwig-Maximilian-University, Munich, Germany; ^4^ Center for Comprehensive Developmental Care (CDeCLMU) at the Social Pediatric Center, Dr. von Hauner Children`s Hospital, Ludwig Maximilian University (LMU) Hospital, Ludwig-Maximilian-University, Munich, Germany; ^5^ Division of Neonatology and Pediatric Intensive Care Medicine, University Medical Center Ulm, Ulm, Germany; ^6^ Department of General Pediatrics and Neonatology, Universities of Giessen and Marburg Lung Center (UGMLC), German Center for Lung Research (DZL), Justus-Liebig-University Giessen, Giessen, Germany; ^7^ Department of Gynecology and Obstetrics, Dr. von Hauner Children’s Hospital, University Hospital, Ludwig-Maximilian-University, Munich, Germany; ^8^ Institute for Medical Microbiology, Justus-Liebig-University Giessen, Giessen, Germany; ^9^ German Center for Cardiovascular Research (DZHK), Partner Site Munich Heart Alliance, Munich, Germany; ^10^ Department of Medicine I, University Hospital, Ludwig Maximilian University, Munich, Germany

**Keywords:** bronchopulmonary dysplasia, monocytes, cytokines, preterm infants, prenatal injury, chronic lung disease

In the published article, there was an error in the Funding statement. The funding statement for the Deutsche Forschungsgemeinschaft (DFG, German Research Foundation) was displayed as “TRR 369”. The correct Funding statement appears below.

**“**The present study was supported by the National Genome Research Network (NGFN; IE-S08T03), the Pneumonia Research Network on Genetic Resistance and Susceptibility for the Evolution of Severe Sepsis (PROGRESS; 01KI07110), the Young Investigator Grant (Helmholtz Association; VH-NG-829), the German Center for Lung Research (DZL; BMBF) and Stiftung AtemWeg (Lung Science Foundation, Project LSS AIRR). Work was supported by the Deutsche Forschungsgemeinschaft (DFG, German Research Foundation) - TRR 359 - Project number 491676693.”

The authors apologize for this error and state that this does not change the scientific conclusions of the article in any way. The original article has been updated.

