# Supplementary material for: Monocyte signature as a predictor of chronic lung disease in the preterm infant
Source: Front Immunol. 2023 Apr 5;14:1112608. doi: 10.3389/fimmu.2023.1112608 (PMC10113536; doi:10.3389/fimmu.2023.1112608)

Supplemental table 1. Predictive Analysis of Microarrays for Bronchopulmonary Dysplasia (BPD (n=13) vs. no BPD (n=9))

	Acc	Symbol	GeneID	Name	Score BPD	Score No BPD
1	AJ223280	LAT	27040	Linker for activation of T cells	-0.0044	0.003
2	AK001814	LOC100505876	100505876	Hypothetical LOC100505876	0.0848	-0.0587
3	AK002039	MRVI1	10335	Murine retrovirus integration site 1 homolog	0.0237	-0.0164
4	AK024496	KLHDC4	54758	Kelch domain containing 4	-0.0108	0.0075
5	AK057700	ATP6V0E2	155066	ATPase, H+ transporting V0 subunit e2	-0.0393	0.0272
6	AL049365	FAM102A	399665	Family with sequence similarity 102, member A	-0.0058	0.004
7	AL162053	FBXO3	26273	F-box protein 3	-0.1301	0.09
8	AL539691				-0.2229	0.1543
9	BE378990	OGDH	4967	Oxoglutarate (alpha-ketoglutarate) dehydrogenase (lipoamide)	-0.0016	0.0011
10	BM741997	SLC25A3	5250	Solute carrier family 25 (mitochondrial carrier; phosphate carrier), member 3	-0.1086	0.0752
11	M77140	GAL	51083	Galanin prepropeptide	0.1592	-0.1102
12	NM_001752	CAT	847	Catalase	0.2576	-0.1783
13	NM_003578	SOAT2	8435	Sterol O-acyltransferase 2	0.0219	-0.0152
14	NM_003832				-0.7084	0.4904
15	NM_003841	TNFRSF10C	8794	Tumor necrosis factor receptor superfamily, member 10c, decoy without an intracellular domain	0.0475	-0.0329
16	NM_004049	BCL2A1	597	BCL2-related protein A1	0.0506	-0.035
17	NM_004418	DUSP2	1844	Dual specificity phosphatase 2	-0.0628	0.0435
18	NM_005451	PDLIM7	9260	PDZ and LIM domain 7 (enigma)	0.0376	-0.026
19	NM_006230	POLD2	5425	Polymerase (DNA directed), delta 2, regulatory subunit 50kDa	0.016	-0.0111
20	NM_006651	CPLX1	10815	Complexin 1	-0.0174	0.0121
21	NM_006746	SCML1	6322	Sex comb on midleg-like 1 (Drosophila)	-0.0599	0.0415
22	NM_006858	TMED1	11018	Transmembrane emp24 protein transport domain containing 1	-0.0339	0.0234
23	NM_014796				-0.0243	0.0168
24	NM_015987	HEBP1	50865	Heme binding protein 1	0.0651	-0.0451
25	NM_018104				0.1138	-0.0788
26	NM_018427	RRN3	54700	RRN3 RNA polymerase I transcription factor homolog (S. cerevisiae)	-0.0901	0.0624
27	NM_030751	ZEB1	6935	Zinc finger E-box binding homeobox 1	-0.1017	0.0704
28	NM_030807	SLC2A11	66035	Solute carrier family 2 (facilitated glucose transporter), member 11	0.6273	-0.4343

Supplemental Table 2. Predictive analysis of microarrays for severity of Bronchopulmonary dysplasia (BPD mild (n=6), moderate to severe (n=3), no (n=13))

	Acc	Symbol	GeneID	Name	Score Mild BPD	Score Moderate/ Severe BPD	Score No BPD
1	AA318707	S100A9	6280	S100 calcium binding protein A9	0	0.1924	0
2	AB028949				0.1382	0	0
3	AF095735	SARDH	1757	Sarcosine dehydrogenase	-0.0604	0	0
4	AJ223280	LAT	27040	Linker for activation of T cells	-0.2355	0	0.0104
5	AK001143				0.1132	0	0
6	AK001814	LOC100505876	100505876	Hypothetical LOC100505876	0	0	-0.031
7	AK002039	MRVI1	10335	Murine retrovirus integration site 1 homolog	0.0437	0	-0.0055
8	AK024496	KLHDC4	54758	Kelch domain containing 4	-0.0644	0	0
9	AL049274	MAN1C1	57134	Mannosidase, alpha, class 1C, member 1	-0.2246	0	0
10	AL049332				0.476	0	-0.035
11	AL110274				0.0148	0	0
12	AL162053	FBXO3	26273	F-box protein 3	0	0	0.0548
13	AL539691				-0.4014	0	0.1663
14	BM741997	SLC25A3	5250	Solute carrier family 25 (mitochondrial carrier; phosphate carrier), member 3	0	0	0.0391
15	M77140	GAL	51083	Galanin prepropeptide	0.8915	0	-0.2242
16	NM_000275	OCA2	4948	Oculocutaneous albinism II	0.0887	0	0
17	NM_000732	CD3D	915	CD3d molecule, delta (CD3-TCR complex)	-0.0314	0	0
18	NM_000985	RPL17	6139	Ribosomal protein L17	-0.1984	0	0
19	NM_001257	CDH13	1012	Cadherin 13, H-cadherin (heart)	0.1587	0	0
20	NM_001311	CRIP1	1396	Cysteine-rich protein 1 (intestinal)	-0.4211	0	0
21	NM_001585				0.135	0	0
22	NM_001607	ACAA1	30	Acetyl-CoA acyltransferase 1	-0.3296	0	0
23	NM_001730	KLF5	688	Kruppel-like factor 5 (intestinal)	-0.0343	0	0
24	NM_001752	CAT	847	Catalase	0.1385	0	-0.1436
25	NM_001803	CD52	1043	CD52 molecule	-0.0859	0	0
26	NM_002108	HAL	3034	Histidine ammonia-lyase	0.0569	0	0

27	NM_002416	CXCL9	4283	Chemokine (C-X-C motif) ligand 9	0.0567	0	0
28	NM_002570	PCSK6	5046	Proprotein convertase subtilisin/kexin type 6	0.0549	0	0
29	NM_002777	PRTN3	5657	Proteinase 3	0	0.0043	0
30	NM_003578	SOAT2	8435	Sterol O-acyltransferase 2	0.0355	0	0
31	NM_003726	SKAP1	8631	Src kinase associated phosphoprotein 1	-0.0702	0	0
32	NM_003832				-0.4922	0	0.4463
33	NM_003841	TNFRSF10C	8794	Tumor necrosis factor receptor superfamily, member 10c, decoy without an intracellular domain	0.0366	0	-0.0115
34	NM_004049	BCL2A1	597	BCL2-related protein A1	0	0	-0.0119
35	NM_004221	IL32	9235	Interleukin 32	-0.0113	0	0
36	NM_004305	BIN1	274	Bridging integrator 1	-0.0293	0	0
37	NM_004418	DUSP2	1844	Dual specificity phosphatase 2	-0.0415	0	0.0147
38	NM_004427	PHC2	1912	Polyhomeotic homolog 2 (Drosophila)	-0.1094	0	0
39	NM_004545	NDUFB1	4707	NADH dehydrogenase (ubiquinone) 1 beta subcomplex, 1, 7kDa	-0.1981	0	0
40	NM_005091	PGLYRP1	8993	Peptidoglycan recognition protein 1	0	0.5115	0
41	NM_005129				-0.2001	0	0
42	NM_005389	PCMT1	5110	Protein-L-isoaspartate (D-aspartate) O-methyltransferase	-0.0468	0	0
43	NM_005608	PTPRCAP	5790	Protein tyrosine phosphatase, receptor type, C-associated protein	-0.1015	0	0
44	NM_005849	IGSF6	10261	Immunoglobulin superfamily, member 6	0	0.0421	0
45	NM_006308	HSPB3	8988	Heat shock 27kDa protein 3	0.0951	0	0
46	NM_006746	SCML1	6322	Sex comb on midleg-like 1 (Drosophila)	0	0	0.0105
47	NM_007147	ZNF175	7728	Zinc finger protein 175	0.0252	0	0
48	NM_014299	BRD4	23476	Bromodomain containing 4	0.0033	0	0
49	NM_014381	MLH3	27030	MutL homolog 3 (E. coli)	-6,00E-04	0	0
50	NM_014801	PCNXL2	80003	Pecanex-like 2 (Drosophila)	-0.0634	0	0
51	NM_015987	HEBP1	50865	Heme binding protein 1	0	0	-0.0069
52	NM_016735				0.0676	0	0
53	NM_017947	MOCOS	55034	Molybdenum cofactor sulfurase	0	0.1855	0
54	NM_018050	MANSC1	54682	MANSC domain containing 1	0.0107	0	0
55	NM_018104				0	0	-0.0415
56	NM_018356	C5orf22	55322	Chromosome 5 open reading frame 22	0.0338	0	0

57	NM_018427	RRN3	54700	RRN3 RNA polymerase I transcription factor homolog (S. cerevisiae)	-0.3781	0	0.089
58	NM_018457	PRR13	54458	Proline rich 13	-0.2336	0	0
59	NM_020689	SLC24A3	57419	Solute carrier family 24 (sodium/potassium/calcium exchanger), member 3	0.1688	0	0
60	NM_020995	HPR	3250	Haptoglobin-related protein	0	0.1752	0
61	NM_021958	HLX	3142	H2.0-like homeobox	0	0.4944	0
62	NM_022154	SLC39A8	64116	Solute carrier family 39 (zinc transporter), member 8	0	0.0029	0
63	NM_025084				-0.1069	0	0
64	NM_030751	ZEB1	6935	Zinc finger E-box binding homeobox 1	0	0	0.0426
65	NM_030807	SLC2A11	66035	Solute carrier family 2 (facilitated glucose transporter), member 11	0.4322	0	-0.3947
66	NM_031296	RAB33B	83452	RAB33B, member RAS oncogene family	0.0319	0	0
67	NM_031885	BBS2	583	Bardet-Biedl syndrome 2	0.09	0	0
68	NM_032031				-0.0133	0	0
69	NM_032621	BEX2	84707	Brain expressed X-linked 2	-0.0344	0	0
70	NM_052972	LRG1	116844	Leucine-rich alpha-2-glycoprotein 1	0	0.0779	0
71	X02160				-0.0999	0	0

Supplemental Table 3. Genes from overrepresentation analysis predicting BPD
Database for Annotation, Visualization, and Integrated Discovery (DAVID, Gene Ontology Term Biological Process Direct)

Gene Ontology Term	Count	%	PValue	Genes
GO:0030073~insulin secretion	2	8.3%	0.049	CPLX1,GAL
GO:0006955~immune response	3	12.5%	0.079	TNFRSF10C, LAT, ZEB1
GO:0006091~generation of precursor metabolites and energy	2	8.3%	0.081	SLC25A3, OGDH
GO:0008203~cholesterol metabolic process	2	8.3%	0.090	SOAT2, CAT
GO:0006954~inflammatory response	3	12.5%	0.094	TNFRSF10C, LAT, GAL

Supplemental Table 4. Genes from overrepresentation analysis predicting BPD severity (DAVID, Gene Ontology Term Biological Process Direct)

Gene Ontology Term	Count	%	PValue	Genes
GO:0006955~immune response	8	12.7%	0.000	PGLYRP1, IGSF6, IL32, TNFRSF10C, CXCL9, ZEB1, CRIP1, LAT
GO:0006952~defense response	3	4.8%	0.021	PTPRCAP, IL32, CXCL9
GO:0008283~cell proliferation	5	7.9%	0.049	RRN3, OCA1, ZEB1, CRIP1, BIN1

Supplemental Table 5. Biological pathways downstream of differentially regulated transcripts associated with disease severity (Ingenuity Pathway Analysis (IPA), Downstream analysis; positive/negative z-scores: biological function predicted to be activated/decreased)

**Diseases and Biological Functions**	**Mild BPD *vs.* no BPD**	**Moderate/ severe BPD *vs.* no BPD**	**Moderate/ severe BPD *vs.* mild BPD**
**weight loss**	-1.71		
**accumulation of eosinophils**	-1.98		
**accumulation of cells**	-2.42		
**accumulation of leukocytes**	-2.20		
**apoptosis of cancer cells**	-2.23		
**damage of kidney**	-2.22		
**cell death of cancer cells**	-2.22		
**accumulation of granulocytes**	-2.22		
**phagocytosis**		-1.73	
**cell movement of vascular smooth muscle cells**			-2.00
**chemotaxis of cells**	2.66		-1.86
**aggregation of cells**	1.95		-0.73
**branching of neurites**			1.96
**chemotaxis of lymphocytes**	1.83		
**airway hyperresponsiveness**	1.85		
**chemotaxis of leukocytes**	2.28		
**quantity of granulocytes**	2.18		
**quantity of neutrophils**	2.06		
**chemotaxis of mononuclear leukocytes**	2.11		

Supplemental Table 6. Differentially expressed gene transcripts in preterm infants with Bronchopulmonary Dysplasia (BPD, no BPD (n=13), mild BPD (n=6), moderate to severe BPD (n=3))

False discovery rate (FDR; FDRs <= 0.05 depicted in red) and fold change (FC; FC > 2 depicted in bold) were calculated with the Linear model for microarray analysis (LIMMA). ACCN: Genbank Accession number, Gene ID from Entrez Gene ID database, Symbols are Hugo gene symbols obtained with SOURCE.

Symbol	ACCN	Name	Gene ID	FDR mild vs. no BPD	FDR mod./ severe vs. no BPD	FDR mod./ severe vs. mild BPD	FC mild vs. no BPD	FC mod./ severe vs. no BPD	FC mod./ severe vs. mild BPD
GAL	M77140	Galanin prepropeptide	51083	0.000	0.975	0.000	**15.49**	1.14	**-13.59**
CRIP1	NM_001311	Cysteine-rich protein 1 (intestinal)	1396	0.000	0.764	0.000	**-8.53**	1.44	**12.30**
SLC24A3	NM_020689	Solute carrier family 24 (sodium/potassium/calcium exchanger), member 3	57419	0.000	0.997	0.006	**4.10**	-1.01	**-4.16**
CDC14A	NM_003672	CDC14 cell division cycle 14 homolog A (S. cerevisiae)	8556	0.000	0.862	0.003	**2.52**	-1.18	**-2.97**
CDH13	NM_001257	Cadherin 13, H-cadherin (heart)	1012	0.000	0.985	0.008	**4.06**	1.05	**-3.87**
ZNF839	NM_018335	Zinc finger protein 839	55778	0.001	0.978	0.007	**-2.54**	1.06	**2.71**
RARS2	BC010420	Arginyl-tRNA synthetase 2, mitochondrial	57038	0.001	0.860	0.008	**-2.26**	1.20	**2.70**
SSRP1	NM_003146	Structure specific recognition protein 1	6749	0.001	0.985	0.018	**2.31**	-1.03	**-2.38**
MAOB	NM_000898	Monoamine oxidase B	4129	0.001	0.791	0.048	**2.31**	1.20	-1.92
FZD2	NM_001466	Frizzled homolog 2 (Drosophila)	2535	0.010	0.208	0.003	**2.04**	-1.86	**-3.79**
LIMD1	NM_014240	LIM domains containing 1	8994	0.001	1.000	0.024	**2.45**	1.00	**-2.45**
PTPRCAP	NM_005608	Protein tyrosine phosphatase, receptor type, C-associated protein	5790	0.001	0.925	0.017	**-2.58**	1.18	**3.05**
ZNF175	NM_007147	Zinc finger protein 175	7728	0.011	0.266	0.003	**2.01**	-1.80	**-3.61**
GYG2	NM_003918	Glycogenin 2	8908	0.004	0.659	0.007	2.00	-1.34	**-2.67**
BBS2	NM_031885	Bardet-Biedl syndrome 2	583	0.001	0.986	0.026	**2.48**	-1.04	**-2.58**
ACAA1	NM_001607	Acetyl-CoA acyltransferase 1	30	0.004	0.724	0.008	**-3.12**	1.51	**4.72**
GJA8	NM_005267	Gap junction protein, alpha 8, 50kDa	2703	0.002	0.981	0.038	**2.33**	1.05	**-2.21**
RAB32	NM_006834	RAB32, member RAS oncogene family	10981	0.036	0.126	0.003	-1.89	**2.06**	**3.89**
IGDCC3	AF063936	Immunoglobulin superfamily, DCC subclass, member 3	9543	0.003	0.961	0.029	**2.07**	-1.10	**-2.29**

Symbol	ACCN	Name	Gene ID	FDR mild vs. no BPD	FDR mod./ severe vs. no BPD	FDR mod./ severe vs. mild BPD	FC mild vs. no BPD	FC mod./ severe vs. no BPD	FC mod./ severe vs. mild BPD
MCC	NM_002387	Mutated in colorectal cancers	4163	**0.003**	0.993	**0.045**	**2.65**	1.03	**-2.58**
DNAJC6	NM_014787	DnaJ (Hsp40) homolog, subfamily C, member 6	9829	**0.047**	0.191	**0.005**	1.54	-1.66	**-2.56**
DNAH3	AL096732	Dynein, axonemal, heavy chain 3	55567	**0.006**	0.808	**0.022**	**2.14**	-1.31	**-2.80**
MICA	NM_000247	MHC class I polypeptide-related sequence A	10050743 6	**0.028**	0.331	**0.007**	-1.63	1.42	**2.31**
OSBP	NM_002556	Oxysterol binding protein	5007	**0.005**	0.992	**0.036**	1.96	-1.02	**-2.00**
KLF5	NM_001730	Kruppel-like factor 5 (intestinal)	688	**0.023**	0.389	**0.008**	-1.96	1.68	**3.30**
PHC2	NM_004427	Polyhomeotic homolog 2 (Drosophila)	1912	**0.012**	0.587	**0.012**	**-2.29**	1.60	**3.67**
MANSC1	NM_018050	MANSC domain containing 1	54682	**0.008**	0.853	**0.024**	**2.25**	-1.26	**-2.83**
MRPL33	NM_004891	Mitochondrial ribosomal protein L33	9553	**0.010**	0.780	**0.022**	**-2.33**	1.36	**3.17**
CCR8	NM_005201	Chemokine (C-C motif) receptor 8	1237	**0.006**	0.971	**0.038**	**2.13**	-1.09	**-2.32**
ROBO1	NM_133631	Roundabout, axon guidance receptor, homolog 1 (Drosophila)	6091	**0.006**	0.971	**0.038**	**2.06**	-1.09	**-2.25**
RNH1	NM_002939	Ribonuclease/angiogenin inhibitor 1	6050	**0.021**	0.528	**0.013**	**2.05**	-1.63	**-3.33**
FNBP1	AB011126	Formin binding protein 1	23048	**0.008**	0.916	**0.033**	**-2.33**	1.20	**2.79**
DUS2L	NM_017803	Dihydrouridine synthase 2-like, SMM1 homolog (S. cerevisiae)	54920	**0.008**	0.951	**0.038**	**2.22**	-1.14	**-2.53**
NEK7	AL080111	NIMA (never in mitosis gene a)-related kinase 7	140609	**0.011**	0.836	**0.029**	**2.09**	-1.26	**-2.63**
ST8SIA5	NM_013305	ST8 alpha-N-acetyl-neuraminide alpha-2,8-sialyltransferase 5	29906	**0.017**	0.716	**0.026**	**2.03**	-1.39	**-2.83**
TNFRSF1A	NM_001065	Tumor necrosis factor receptor superfamily, member 1A	7132	**0.010**	0.944	**0.040**	**2.02**	-1.14	**-2.30**
PCSK6	NM_002570	Proprotein convertase subtilisin/kexin type 6	5046	**0.010**	0.913	**0.040**	**2.46**	-1.25	**-3.06**
PFN2	NM_053024	Profilin 2	5217	**0.025**	0.631	**0.022**	1.98	-1.48	**-2.94**
RNASE1	NM_002933	Ribonuclease, RNase A family, 1 (pancreatic)	6035	**0.021**	0.733	**0.029**	1.99	-1.37	**-2.71**
IL1RL2	NM_003854	Interleukin 1 receptor-like 2	8808	**0.019**	0.814	**0.035**	1.69	-1.21	**-2.04**
NDUFB1	NM_004545	NADH dehydrogenase (ubiquinone) 1 beta subcomplex, 1, 7kDa	4707	**0.025**	0.732	**0.031**	**-3.06**	1.62	**4.95**
PDGFRB	NM_002609	Platelet-derived growth factor receptor, beta polypeptide	5159	**0.039**	0.581	**0.028**	1.57	-1.35	**-2.12**
RPL36	NM_015414	Ribosomal protein L36	25873	**0.034**	0.694	**0.033**	**-2.04**	1.43	**2.91**

Symbol	ACCN	Name	Gene ID	FDR mild vs. no BPD	FDR mod./ severe vs. no BPD	FDR mod./ severe vs. mild BPD	FC mild vs. no BPD	FC mod./ severe vs. no BPD	FC mod./ severe vs. mild BPD
ZNF274	NM_016325	Zinc finger protein 274	10782	**0.045**	0.611	**0.031**	-1.74	1.45	**2.52**
SPRY3	AA933967	Sprouty homolog 3 (Drosophila)	10251	**0.050**	0.694	**0.038**	1.65	-1.31	**-2.17**
EHHADH	NM_001966	Enoyl-CoA, hydratase/3-hydroxyacyl CoA dehydrogenase	1962	**0.037**	0.777	**0.045**	1.93	-1.37	**-2.64**
SLC2A11	NM_030807	Solute carrier family 2 (facilitated glucose transporter), member 11	66035	**0.000**	**0.014**	0.888	**6.93**	**6.15**	-1.13
TMED1	NM_006858	Transmembrane emp24 protein transport domain containing 1	11018	**0.003**	**0.041**	0.657	-1.75	**-2.05**	-1.17
TECPR1	AB037779	Tectonin beta-propeller repeat containing 1	25851	**0.023**	**0.007**	0.316	-1.60	**-2.21**	-1.37
ACTN2	NM_001103	Actinin, alpha 2	88	**0.036**	**0.033**	0.455	**2.16**	**3.28**	1.52
OR2S2	NM_019897	Olfactory receptor, family 2, subfamily S, member 2	56656	**0.000**	0.284	0.141	**2.87**	1.67	-1.71
SLC25A3	BM741997	Solute carrier family 25 (mitochondrial carrier; phosphate carrier), member 3	5250	**0.001**	0.191	0.413	**-4.79**	**-2.77**	1.73
HSPB3	NM_006308	Heat shock 27kDa protein 3	8988	**0.001**	0.862	0.056	**2.64**	1.22	**-2.17**
SLC7A8	NM_012244	Solute carrier family 7 (amino acid transporter, L-type), member 8	23428	**0.001**	0.875	0.050	**3.02**	1.23	**-2.46**
MRVI1	AK002039	Murine retrovirus integration site 1 homolog	10335	**0.001**	0.584	0.154	**2.08**	1.33	-1.57
RRN3	NM_018427	RRN3 RNA polymerase I transcription factor homolog (S. cerevisiae)	54700	**0.001**	0.928	0.063	**-3.96**	-1.25	**3.18**
MAN1C1	AL049274	Mannosidase, alpha, class 1C, member 1	57134	**0.003**	0.981	0.057	**-2.78**	-1.07	**2.60**
PCNXL2	NM_014801	Pecanex-like 2 (Drosophila)	80003	**0.003**	0.961	0.076	**-2.91**	-1.15	**2.53**
MALL	U17077	Mal, T-cell differentiation protein-like	7851	**0.003**	0.546	0.397	**2.12**	1.50	-1.41
BEX2	NM_032621	Brain expressed X-linked 2	84707	**0.004**	0.997	0.057	**-2.13**	1.01	**2.15**
HIVEP2	X65644	Human immunodeficiency virus type I enhancer binding protein 2	3097	**0.004**	0.987	0.057	**-2.24**	-1.03	**2.16**
TLR3	NM_003265	Toll-like receptor 3	7098	**0.004**	0.955	0.101	**2.12**	1.12	-1.89
SCML1	NM_006746	Sex comb on midleg-like 1 (Drosophila)	6322	**0.006**	0.256	0.822	**-2.35**	**-2.08**	1.13
LGALS3	NM_002306	Lectin, galactoside-binding, soluble, 3	3958	**0.006**	0.389	0.467	**-2.50**	-1.75	1.43
OCA2	NM_000275	Oculocutaneous albinism II	4948	**0.006**	0.999	0.067	**2.46**	1.01	**-2.44**
DUSP8	NM_004420	Dual specificity phosphatase 8	1850	**0.005**	0.939	0.131	**2.59**	1.19	**-2.18**
DHRS2	NM_005794	Dehydrogenase/reductase (SDR family) member 2	10202	**0.007**	0.994	0.057	**2.67**	-1.02	**-2.73**

Symbol	ACCN	Name	Gene ID	FDR mild vs. no BPD	FDR mod./ severe vs. no BPD	FDR mod./ severe vs. mild BPD	FC mild vs. no BPD	FC mod./ severe vs. no BPD	FC mod./ severe vs. mild BPD
CAT	NM_001752	Catalase	847	0.007	0.389	0.532	**6.67**	**3.42**	-1.95
FBXL2	NM_012157	F-box and leucine-rich repeat protein 2	25827	0.007	0.331	0.626	**-2.19**	-1.76	1.24
CXCL9	NM_002416	Chemokine (C-X-C motif) ligand 9	4283	0.006	0.983	0.086	**2.94**	1.07	**-2.75**
ACVR1B	NM_004302	Activin A receptor, type IB	91	0.006	0.983	0.091	**2.84**	1.07	**-2.66**
OLFM2	NM_058164	Olfactomedin 2	93145	0.006	0.946	0.121	**2.15**	1.13	-1.90
TNFRSF10C	NM_003841	Tumor necrosis factor receptor superfamily, member 10c, decoy without an intracellular domain	8794	0.006	0.640	0.342	**2.84**	1.62	-1.75
DHX15	NM_001358	DEAH (Asp-Glu-Ala-His) box polypeptide 15	1665	0.008	1.000	0.076	**2.21**	-1.00	**-2.21**
FBXO3	AL162053	F-box protein 3	26273	0.018	0.124	0.883	**-2.07**	**-2.24**	-1.08
AHCYL1	NM_006621	Adenosylhomocysteinase-like 1	10768	0.008	0.979	0.105	**2.25**	1.07	**-2.09**
FAM102A	AL049365	Family with sequence similarity 102, member A	399665	0.007	0.702	0.295	**-2.70**	-1.50	1.80
MGAM	NM_004668	Maltase-glucoamylase (alpha-glucosidase)	8972	0.010	0.320	0.669	**2.64**	**2.05**	-1.29
RAB33B	NM_031296	RAB33B, member RAS oncogene family	83452	0.007	0.925	0.172	**2.28**	1.18	-1.92
ABCB11	NM_003742	ATP-binding cassette, sub-family B (MDR/TAP), member 11	8647	0.008	0.832	0.208	**2.26**	1.27	-1.78
PRR13	NM_018457	Proline rich 13	54458	0.012	0.971	0.056	**-3.37**	1.17	**3.94**
RPL17	NM_000985	Ribosomal protein L17	6139	0.012	0.962	0.053	**-3.88**	1.23	**4.79**
ZNF157	NM_003446	Zinc finger protein 157	7712	0.011	0.986	0.074	**2.28**	-1.04	**-2.38**
CEP192	NM_032142	Centrosomal protein 192kDa	55125	0.010	0.981	0.111	**2.39**	1.08	**-2.21**
PROC	NM_000312	Protein C (inactivator of coagulation factors Va and VIIIa)	5624	0.008	0.925	0.202	**2.55**	1.23	**-2.07**
MAPK8IP2	NM_012324	Mitogen-activated protein kinase 8 interacting protein 2	23542	0.010	0.587	0.394	**2.69**	1.65	-1.63
CELSR3	NM_001407	Cadherin, EGF LAG seven-pass G-type receptor 3 (flamingo homolog, Drosophila)	1951	0.012	0.981	0.071	**2.14**	-1.06	**-2.28**
DIO3	NM_001362	Deiodinase, iodothyronine, type III	1735	0.010	0.956	0.156	**2.83**	1.18	**-2.39**
ANO3	NM_031418	Anoctamin 3	63982	0.010	0.883	0.205	**2.25**	1.23	-1.83
LAT	AJ223280	Linker for activation of T cells	27040	0.010	0.975	0.135	**-3.15**	-1.13	**2.78**
CLCN5	NM_000084	Chloride channel 5	1184	0.010	0.857	0.222	**2.26**	1.26	-1.79
POLD2	NM_006230	Polymerase (DNA directed), delta 2, regulatory subunit 50kDa	5425	0.010	0.659	0.405	**2.32**	1.51	-1.53

Symbol	ACCN	Name	Gene ID	FDR mild vs. no BPD	FDR mod./ severe vs. no BPD	FDR mod./ severe vs. mild BPD	FC mild vs. no BPD	FC mod./ severe vs. no BPD	FC mod./ severe vs. mild BPD
SEMA5A	NM_003966	Sema domain, seven thrombospondin repeats (type 1 and type 1-like), transmembrane domain (TM) and short cytoplasmic domain, (semaphorin) 5A	9037	**0.011**	0.925	0.189	**2.95**	1.26	**-2.35**
MCAT	AL359403	Malonyl CoA:ACP acyltransferase (mitochondrial)	27349	**0.021**	0.887	0.055	1.84	-1.18	**-2.17**
ATP6V0E2	AK057700	ATPase, H+ transporting V0 subunit e2	155066	**0.021**	0.289	0.884	**-2.09**	-1.93	1.08
VSIG4	NM_007268	V-set and immunoglobulin domain containing 4	11326	**0.012**	0.985	0.126	**2.21**	1.05	**-2.11**
KLHDC4	AK024496	Kelch domain containing 4	54758	**0.011**	0.855	0.256	**-2.76**	-1.36	**2.04**
GRIA3	NM_007325	Glutamate receptor, ionotrophic, AMPA 3	2892	**0.011**	0.693	0.409	**-2.54**	-1.57	1.62
LDHD	AA203281	Lactate dehydrogenase D	197257	**0.012**	0.993	0.129	**2.06**	1.03	**-2.00**
PCMT1	NM_005389	Protein-L-isoaspartate (D-aspartate) O-methyltransferase	5110	**0.022**	0.887	0.056	**-2.17**	1.25	**2.71**
CERK	NM_022766	Ceramide kinase	64781	**0.012**	0.970	0.173	**2.45**	1.14	**-2.15**
ASGR1	NM_001671	Asialoglycoprotein receptor 1	432	**0.012**	0.792	0.296	**2.79**	1.44	-1.93
C12orf5	NM_020375	Chromosome 12 open reading frame 5	57103	**0.012**	0.708	0.353	**-2.41**	-1.46	1.66
BID	NM_001196	BH3 interacting domain death agonist	637	**0.019**	0.978	0.077	**-2.42**	1.11	**2.67**
CST4	NM_001899	Cystatin S	1472	**0.012**	0.780	0.297	**2.58**	1.41	-1.83
DNAJC12	NM_021800	DnaJ (Hsp40) homolog, subfamily C, member 12	56521	**0.012**	0.925	0.202	**2.04**	1.17	-1.75
TMBIM1	NM_022152	Transmembrane BAX inhibitor motif containing 1	64114	**0.018**	0.981	0.092	**2.62**	-1.09	**-2.86**
CFI	NM_000204	Complement factor I	3426	**0.020**	0.943	0.074	1.96	-1.16	**-2.27**
ABCC8	NM_000352	ATP-binding cassette, sub-family C (CFTR/MRP), member 8	6833	**0.025**	0.925	0.062	1.80	-1.14	**-2.06**
SLC34A1	NM_003052	Solute carrier family 34 (sodium phosphate), member 1	6569	**0.024**	0.899	0.066	**2.06**	-1.23	**-2.54**
MMP7	NM_002423	Matrix metallopeptidase 7 (matrilysin, uterine)	4316	**0.019**	0.993	0.100	**2.52**	-1.03	**-2.60**
NUP210	AB020713	Nucleoporin 210kDa	23225	**0.018**	1.000	0.118	**2.27**	-1.00	**-2.28**
GNAO1	AL512686	Guanine nucleotide binding protein (G protein), alpha activating activity polypeptide O	2775	**0.029**	0.886	0.063	1.96	-1.23	**-2.41**
SOAT2	NM_003578	Sterol O-acyltransferase 2	8435	**0.015**	0.780	0.354	**2.68**	1.48	-1.81
HLA-G	NM_002127	Major histocompatibility complex, class I, G	3135	**0.025**	0.978	0.080	**-2.51**	1.10	**2.76**
KIAA1109	AB029032	KIAA1109	84162	**0.016**	0.922	0.237	**2.46**	1.24	-1.98

Symbol	ACCN	Name	Gene ID	FDR mild vs. no BPD	FDR mod./ severe vs. no BPD	FDR mod./ severe vs. mild BPD	FC mild vs. no BPD	FC mod./ severe vs. no BPD	FC mod./ severe vs. mild BPD
MLH3	NM_014381	MutL homolog 3 (E. coli)	27030	**0.025**	0.960	0.086	**-2.64**	1.20	**3.18**
GPR17	NM_005291	G protein-coupled receptor 17	2840	**0.018**	0.955	0.209	**2.58**	1.19	**-2.17**
LDOC1	NM_012317	Leucine zipper, down-regulated in cancer 1	23641	**0.020**	0.981	0.166	**-2.41**	-1.08	**2.23**
AAK1	AF090101	AP2 associated kinase 1	22848	**0.020**	0.654	0.450	**2.32**	1.54	-1.51
GPR183	NM_004951	G protein-coupled receptor 183	1880	**0.022**	0.516	0.624	**-3.15**	**-2.16**	1.46
ZNF395	NM_018660	Zinc finger protein 395	55893	**0.023**	0.985	0.109	1.92	-1.05	**-2.01**
PRAME	NM_006115	Preferentially expressed antigen in melanoma	23532	**0.022**	0.993	0.122	**2.25**	-1.03	**-2.32**
ZFP36	NM_003407	Zinc finger protein 36, C3H type, homolog (mouse)	7538	**0.025**	0.985	0.100	**-2.77**	1.07	**2.97**
KIF22	NM_007317	Kinesin family member 22	3835	**0.021**	0.997	0.148	**2.35**	1.01	**-2.32**
HAL	NM_002108	Histidine ammonia-lyase	3034	**0.020**	0.971	0.207	**2.63**	1.15	**-2.29**
CXorf27	AF049615	Chromosome X open reading frame 27	25763	**0.026**	0.498	0.711	**2.04**	1.68	-1.21
NNMT	NM_006169	Nicotinamide N-methyltransferase	4837	**0.035**	0.915	0.081	**2.06**	-1.23	**-2.53**
ACTR6	NM_022496	ARP6 actin-related protein 6 homolog (yeast)	64431	**0.022**	0.607	0.623	**-2.39**	-1.75	1.37
CTDSP2	AF000152	CTD (carboxy-terminal domain, RNA polymerase II, polypeptide A) small phosphatase 2	10106	**0.049**	0.824	0.064	**2.09**	-1.37	**-2.87**
SARDH	AF095735	Sarcosine dehydrogenase	1757	**0.034**	0.943	0.093	**-2.83**	1.27	**3.60**
DHX32	NM_018180	DEAH (Asp-Glu-Ala-His) box polypeptide 32	55760	**0.039**	0.875	0.083	1.89	-1.27	**-2.40**
CD52	NM_001803	CD52 molecule	1043	**0.027**	0.992	0.147	**-2.75**	1.04	**2.86**
PADI2	AB023211	Peptidyl arginine deiminase, type II	11240	**0.044**	0.925	0.080	1.81	-1.16	**-2.10**
SCG3	AF453583	Secretogranin III	29106	0.068	**0.000**	**0.000**	1.53	**5.44**	**3.55**
HPR	NM_020995	Haptoglobin-related protein	3250	0.974	**0.001**	**0.001**	-1.03	**19.73**	**20.31**
MAP4K3	NM_003618	Mitogen-activated protein kinase kinase kinase kinase 3	8491	0.220	**0.001**	**0.022**	1.68	**6.67**	**3.96**
GNG11	BC009709	Guanine nucleotide binding protein (G protein), gamma 11	2791	0.237	**0.001**	**0.029**	-1.35	**-2.98**	**-2.21**
ZNF576	NM_024327	Zinc finger protein 576	79177	0.785	**0.003**	**0.003**	-1.09	**2.68**	**2.91**
KLKB1	NM_000892	Kallikrein B, plasma (Fletcher factor) 1	3818	0.690	**0.005**	**0.003**	-1.09	**2.03**	**2.20**
ZBTB40	NM_014870	Zinc finger and BTB domain containing 40	9923	0.906	**0.003**	**0.006**	1.05	**3.59**	**3.41**

Symbol	ACCN	Name	Gene ID	FDR mild vs. no BPD	FDR mod./ severe vs. no BPD	FDR mod./ severe vs. mild BPD	FC mild vs. no BPD	FC mod./ severe vs. no BPD	FC mod./ severe vs. mild BPD
FKBP14	NM_017946	FK506 binding protein 14, 22 kDa	55033	0.698	**0.006**	**0.004**	1.13	**-3.04**	**-3.43**
HLX	NM_021958	H2.0-like homeobox	3142	0.845	**0.005**	**0.005**	-1.10	**4.55**	**5.01**
SRD5A2	NM_000348	Steroid-5-alpha-reductase, alpha polypeptide 2 (3-oxo-5 alpha-steroid delta 4-dehydrogenase alpha 2)	6716	0.523	**0.003**	**0.017**	1.24	**3.87**	**3.11**
CDA	NM_001785	Cytidine deaminase	978	0.384	**0.015**	**0.004**	-1.40	**4.45**	**6.25**
PGLYRP1	NM_005091	Peptidoglycan recognition protein 1	8993	0.553	**0.014**	**0.005**	-1.37	**5.76**	**7.87**
ATP7B	NM_000053	ATPase, Cu++ transporting, beta polypeptide	540	0.731	**0.014**	**0.007**	1.09	**-2.37**	**-2.58**
SCNN1G	NM_001039	Sodium channel, nonvoltage-gated 1, gamma	6340	0.323	**0.041**	**0.007**	-1.35	**2.74**	**3.70**
DUSP13	NM_016364	Dual specificity phosphatase 13	51207	0.780	**0.015**	**0.013**	-1.13	**3.04**	**3.43**
WDR5	NM_017588	WD repeat domain 5	11091	0.719	**0.014**	**0.031**	-1.09	**-2.26**	**-2.07**
DNAJB1	NM_006145	DnaJ (Hsp40) homolog, subfamily B, member 1	3337	0.527	**0.033**	**0.010**	-1.24	**3.12**	**3.86**
SLC39A8	NM_022154	Solute carrier family 39 (zinc transporter), member 8	64116	0.529	**0.042**	**0.013**	-1.25	**2.69**	**3.36**
IGSF6	NM_005849	Immunoglobulin superfamily, member 6	10261	0.616	**0.045**	**0.018**	-1.31	**4.06**	**5.33**
HOXD9	NM_014213	Homeobox D9	3235	0.815	**0.026**	**0.040**	1.10	**2.84**	**2.58**
ZHX1	NM_007222	Zinc fingers and homeoboxes 1	11244	0.844	**0.032**	**0.046**	1.07	**2.33**	**2.18**
BATF	NM_006399	Basic leucine zipper transcription factor, ATF-like	10538	0.809	**0.043**	**0.029**	-1.09	**2.45**	**2.68**
RAD51L3	NM_002878	RAD51-like 3 (S. cerevisiae)	5892	0.849	**0.043**	**0.029**	1.07	**-2.28**	**-2.43**
ETNK1	NM_018638	Ethanolamine kinase 1	55500	0.994	**0.037**	**0.036**	1.00	**-2.73**	**-2.74**
RNF24	NM_007219	Ring finger protein 24	11237	0.843	**0.047**	**0.033**	-1.11	**3.00**	**3.31**
WASL	NM_003941	Wiskott-Aldrich syndrome-like	8976	0.989	**0.042**	**0.040**	1.01	**-2.26**	**-2.28**
FLT1	NM_002019	Fms-related tyrosine kinase 1 (vascular endothelial growth factor/vascular permeability factor receptor)	2321	0.094	0.191	**0.010**	1.37	-1.60	**-2.19**
H1FX	NM_006026	H1 histone family, member X	8971	0.302	0.080	**0.013**	1.24	-1.62	**-2.02**
KIAA1199	AB033025	KIAA1199	57214	0.067	0.319	**0.014**	1.88	**-2.04**	**-3.84**
PTP4A1	NM_003463	Protein tyrosine phosphatase type IVA, member 1	7803	0.065	0.389	**0.020**	1.98	-1.97	**-3.90**
SERPINA1	NM_000295	Serpin peptidase inhibitor, clade A (alpha-1 antiproteinase, antitrypsin), member 1	5265	0.215	0.185	**0.016**	-1.81	**3.00**	**5.44**
GPR116	AB018301	G protein-coupled receptor 116	221395	0.360	0.114	**0.018**	-1.28	1.98	**2.55**

Symbol	ACCN	Name	Gene ID	FDR mild vs. no BPD	FDR mod./ severe vs. no BPD	FDR mod./ severe vs. mild BPD	FC mild vs. no BPD	FC mod./ severe vs. no BPD	FC mod./ severe vs. mild BPD
PRKCI	NM_002740	Protein kinase C, iota	5584	0.692	0.051	**0.025**	1.10	**-2.04**	**-2.25**
AK2	NM_001625	Adenylate kinase 2	204	0.094	0.366	**0.025**	1.62	-1.79	**-2.90**
MRPS10	NM_018141	Mitochondrial ribosomal protein S10	55173	0.051	0.587	**0.033**	-1.54	1.38	**2.12**
RABL3	AK025772	RAB, member of RAS oncogene family-like 3	285282	0.050	0.563	**0.034**	-1.53	1.42	**2.17**
NOL10	NM_024894	Nucleolar protein 10	79954	0.182	0.256	**0.024**	-1.44	1.83	**2.64**
NIP7	NM_016101	Nuclear import 7 homolog (S. cerevisiae)	51388	0.493	0.104	**0.026**	1.20	**-2.02**	**-2.41**
TFB1M	NM_016020	Transcription factor B1, mitochondrial	51106	0.199	0.256	**0.024**	-1.63	**2.26**	**3.69**
HIBADH	AK025558	3-hydroxyisobutyrate dehydrogenase	11112	0.315	0.186	**0.026**	1.33	-1.88	**-2.49**
CD63	NM_001780	CD63 molecule	967	0.085	0.497	**0.033**	-1.86	1.74	**3.25**
HEBP2	NM_014320	Heme binding protein 2	23593	0.096	0.480	**0.031**	-1.88	1.80	**3.38**
GPR160	NM_014373	G protein-coupled receptor 160	26996	0.069	0.559	**0.036**	-1.87	1.67	**3.12**
SPOP	NM_003563	Speckle-type POZ protein	8405	0.064	0.589	**0.036**	1.91	-1.63	**-3.11**
DCTN2	NM_006400	Dynactin 2 (p50)	10540	0.482	0.115	**0.031**	1.20	-1.80	**-2.16**
FUBP1	AL049951	Far upstream element (FUSE) binding protein 1	8880	0.050	0.699	**0.040**	**-2.13**	1.55	**3.30**
ZNF256	NM_005773	Zinc finger protein 256	10172	0.070	0.587	**0.036**	-1.78	1.57	**2.79**
ID4	BC014941	Inhibitor of DNA binding 4, dominant negative helix-loop-helix protein	3400	0.258	0.253	**0.030**	1.43	**-2.02**	**-2.90**
PDHA2	NM_005390	Pyruvate dehydrogenase (lipoamide) alpha 2	5161	0.075	0.562	**0.036**	**2.15**	-1.87	**-4.03**
PI16	AI524085	Peptidase inhibitor 16	221476	0.061	0.657	**0.038**	1.56	-1.35	**-2.11**
FCGR3B	J04162	Fc fragment of IgG, low affinity IIIb, receptor (CD16b)	2215	0.068	0.619	**0.038**	**-3.09**	**2.08**	**6.44**
CYP27A1	NM_000784	Cytochrome P450, family 27, subfamily A, polypeptide 1	1593	0.094	0.527	**0.036**	-1.87	1.78	**3.34**
CDCA7L	NM_018719	Cell division cycle associated 7-like	55536	0.667	0.110	**0.038**	-1.11	1.91	**2.13**
LGALS12	NM_033101	Lectin, galactoside-binding, soluble, 12	85329	0.115	0.492	**0.036**	-1.96	1.95	**3.84**
P2RY6	NM_004154	Pyrimidinergic receptor P2Y, G-protein coupled, 6	5031	0.711	0.110	**0.038**	-1.15	**2.34**	**2.69**
ACAT1	NM_000019	Acetyl-CoA acetyltransferase 1	38	0.347	0.228	**0.033**	-1.30	1.75	**2.27**
SMAD1	NM_005900	SMAD family member 1	4086	0.090	0.553	**0.040**	1.56	-1.48	**-2.31**

Symbol	ACCN	Name	Gene ID	FDR mild vs. no BPD	FDR mod./ severe vs. no BPD	FDR mod./ severe vs. mild BPD	FC mild vs. no BPD	FC mod./ severe vs. no BPD	FC mod./ severe vs. mild BPD
C18orf8	NM_013326	Chromosome 18 open reading frame 8	29919	0.070	0.668	**0.043**	1.68	-1.42	**-2.38**
CEBPB	NM_005194	CCAAT/enhancer binding protein (C/EBP), beta	1051	0.113	0.506	**0.038**	**-2.14**	**2.09**	**4.48**
SPAG6	NM_012443	Sperm associated antigen 6	9576	0.394	0.200	**0.034**	-1.35	**2.17**	**2.93**
KHNYN	AB002321	KH and NYN domain containing	23351	0.282	0.284	**0.034**	1.41	-1.87	**-2.63**
TCTE3	AK023627	T-complex-associated-testis-expressed 3	6991	0.145	0.453	**0.038**	-1.66	1.81	**3.01**
FLOT1	NM_005803	Flotillin 1	10211	0.093	0.608	**0.043**	**-2.31**	1.89	**4.37**
TMEM208	NM_014187	Transmembrane protein 208	29100	0.782	0.110	**0.045**	-1.16	**3.02**	**3.51**
NCF2	NM_000433	Neutrophil cytosolic factor 2	4688	0.082	0.652	**0.048**	-1.85	1.57	**2.91**
LOC283174	AL133591	Hypothetical LOC283174	283174	0.081	**0.015**	0.221	1.62	**2.70**	1.67
PRTN3	NM_002777	Proteinase 3	5657	0.321	**0.015**	0.068	1.40	**3.87**	**2.77**
SLC29A1	NM_004955	Solute carrier family 29 (nucleoside transporters), member 1	2030	0.147	**0.026**	0.220	-1.46	**-2.35**	-1.61
ZEB1	NM_030751	Zinc finger E-box binding homeobox 1	6935	0.064	**0.043**	0.404	**-2.32**	**-4.20**	-1.81
HMOX1	NM_002133	Heme oxygenase (decycling) 1	3162	0.441	**0.022**	0.098	-1.25	**-2.39**	-1.92
STAT2	NM_005419	Signal transducer and activator of transcription 2, 113kDa	6773	0.200	**0.033**	0.201	1.41	**2.43**	1.72
TULP3	NM_003324	Tubby like protein 3	7289	0.781	**0.028**	0.050	1.11	**2.66**	**2.39**
SPTBN1	NM_003128	Spectrin, beta, non-erythrocytic 1	6711	0.585	**0.036**	0.090	1.18	**2.28**	1.94
LRG1	NM_052972	Leucine-rich alpha-2-glycoprotein 1	116844	0.521	**0.037**	0.109	1.39	**4.22**	**3.05**
YWHAE	NM_006761	Tyrosine 3-monooxygenase/tryptophan 5-monooxygenase activation protein, epsilon polypeptide	7531	0.225	**0.045**	0.284	1.46	**2.37**	1.62
MX1	NM_002462	Myxovirus (influenza virus) resistance 1, interferon-inducible protein p78 (mouse)	4599	0.805	**0.043**	0.068	1.14	**3.37**	**2.97**
IMPA2	NM_014214	Inositol(myo)-1(or 4)-monophosphatase 2	3613	0.972	**0.045**	0.061	1.02	**2.56**	**2.51**
DEPTOR	NM_022783	DEP domain containing MTOR-interacting protein	64798	0.508	**0.047**	0.139	1.25	**2.41**	1.94

Supplemental Table 7. Cytokine upstream regulators (Ingenuity Pathway analysis (IPA)) predicted to be regulated in preterm infants with Bronchopulmonary Dysplasia at birth (Predictive Analysis of Microarrays, threshold=2.2); positive/negative z-scores: biological function predicted to be activated (red)/decreased (blue); asterisks indicate significance level for enrichment: * $p < 0.05$, ** $p < 0.01$, *** $p < 0.001$.

	Z-scores					
**Upstream regulators**	**Mild BPD – no BPD**		**mod. /sev. BPD – no BPD**		**mod. /sev. BPD – mild BPD**	
**TNF**	**0.29**	**	**1.97**	**	**1.50**	**
**Csf**	**0.00**	**	**0.00**	**	**0.00**	**
**Interferon alpha**	**-0.74**	**	**1.23**	**	**0.00**	**
**IL32**	**0.00**	*	**0.00**	*	**0.00**	*
**CCL19**	**0.00**	*	**0.00**	*	**0.00**	*
**CCL21**	**0.00**	*	**0.00**	*	**0.00**	*
**IL4**	**0.00**	*	**0.00**	*	**0.00**	*
**IL6**	**0.45**	*	**1.34**	*	**0.45**	*
**IL12 (complex)**	**0.00**	*	**0.00**	*	**0.00**	*
**CCL3L1/CCL3L3**	**0.00**	*	**0.00**	*	**0.00**	*
**Ifn gamma**	**0.00**	*	**0.00**	*	**0.00**	*
**IL3**	**0.00**	*	**0.00**	*	**0.00**	*

Supplemental Table 8. Cytokine upstream regulators (Ingenuity Pathway analysis (IPA)) predicted to be regulated in preterm infants with BPD at birth (Differential gene expression analysis): positive/negative z-scores: biological function predicted to be activated (red)); asterisks indicate significance level for enrichment: * p < 0.05, ** p < 0.01, *** p < 0.001)

Upstream regulators	mild vs no BPD		moderate/severe BPD vs. no BPD		moderate/severe vs. mild BPD
IL2	1.80				
TNF	1.66	**			
IL6	1.52	*	0.00	*	
IL10	1.21	*			
IFN alpha/beta	1.13	**			
IFNG	0.56	*			
CCL19	0.00	**			
CCL8	0.00	*			
CXCL9	0.00	*			
Ifn gamma	0.00	**			
IFNE	0.00	**			
IFNK	0.00	*			
IFNW1	0.00	**			
Mac	0.00	*			
TSLP	0.00	*			
CSF3			0.00	*	
EBI3			0.00	**	
IFN Beta			0.00	*	
IFNA1/IFNA13			0.00	*	
IFNA10			0.00	*	
IFNA14			0.00	*	
IFNA17			0.00	*	
IFNA21			0.00	*	
IFNA4			0.00	*	

IFNA5	0.00	*		
IFNA6	0.00	*		
IFNA7	0.00	*		
IFNA8	0.00	*		
IL27	0.00	**		
IL5			2.00	
IL8	0.00	*		
WNT1			0.00	*

Supplemental Figure 1. Transcriptome profile predicting Bronchopulmonary Dysplasia (BPD) by hierarchical clustering on scaled data with Euclidean distance measure and Ward's Linkage clustering method (Predictive Analysis of Microarrays). Disease grades are color coded: no BPD blue, mild BPD red, moderate / severe BPD green.

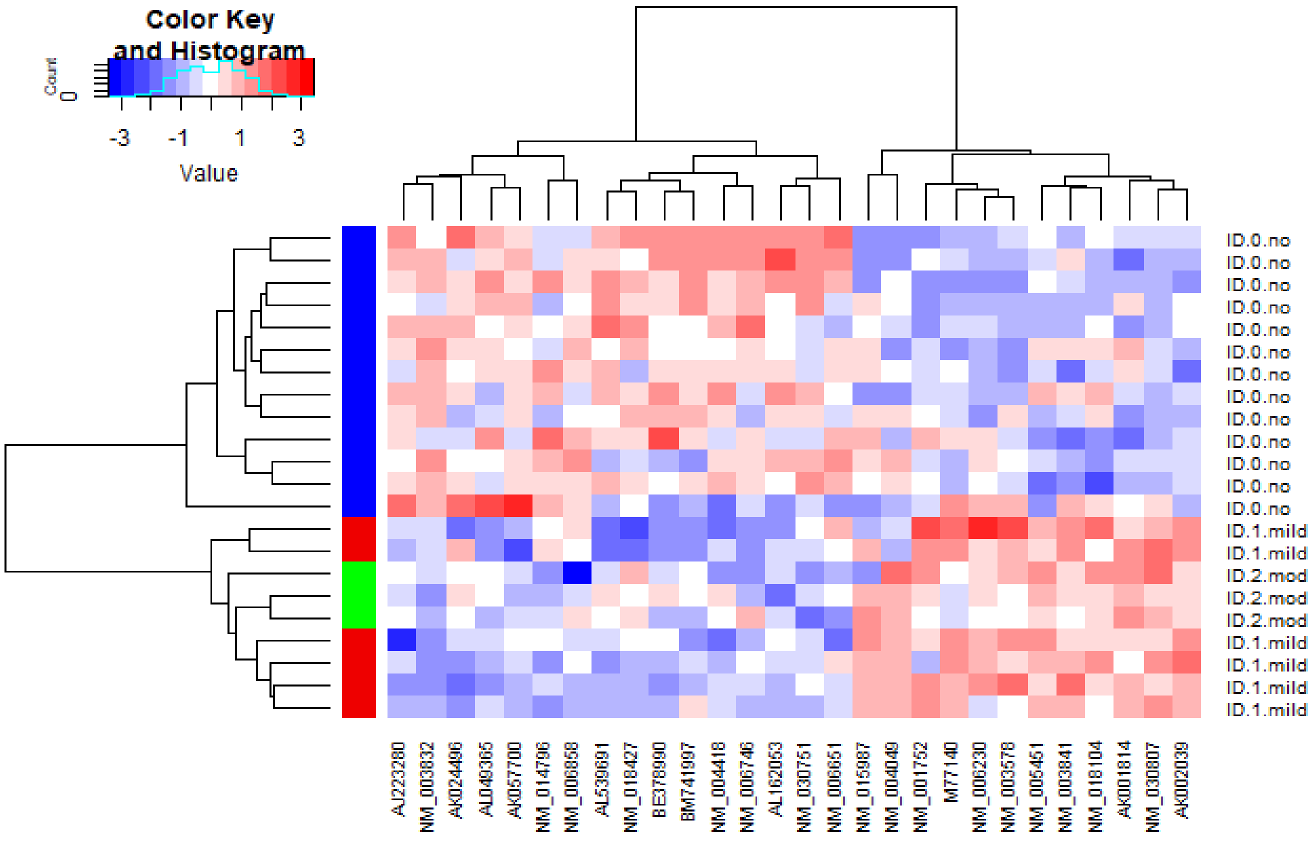

Supplemental Figure 2. Transcriptome profiles predicting Bronchopulmonary Dysplasia (BPD) severity by hierarchical clustering on scaled data **with** Euclidean distance measure and Ward's Linkage clustering method (no BPD blue, mild BPD red, moderate / severe BPD green).

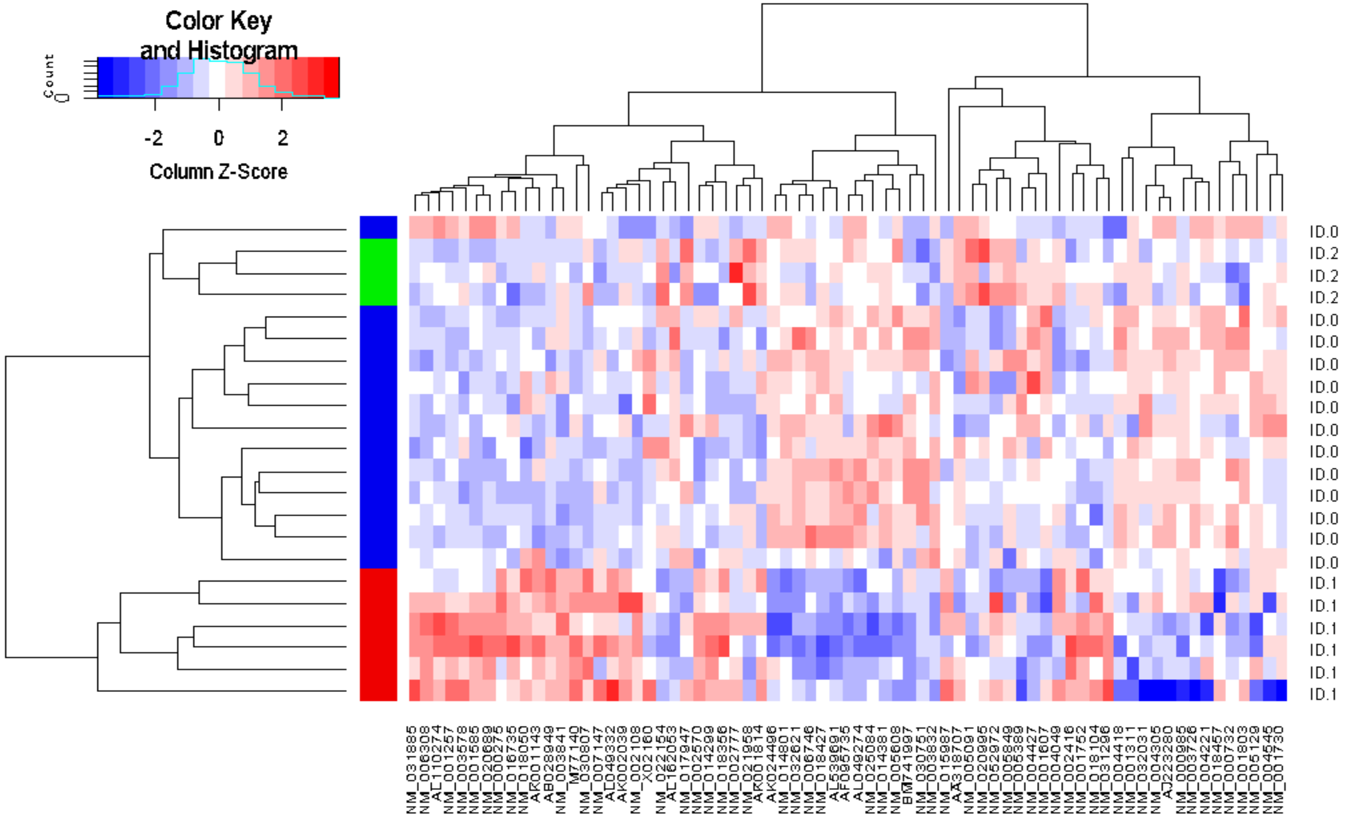

Supplemental Figure 3. Regulator effector networks in infants with mild Bronchopulmonary Dysplasia (BPD).

Regulator effector networks of differentially regulated genes (mild BPD vs. no BPD) derived from Ingenuity Pathway analysis of the transcripts that differentiate between BPD severity.

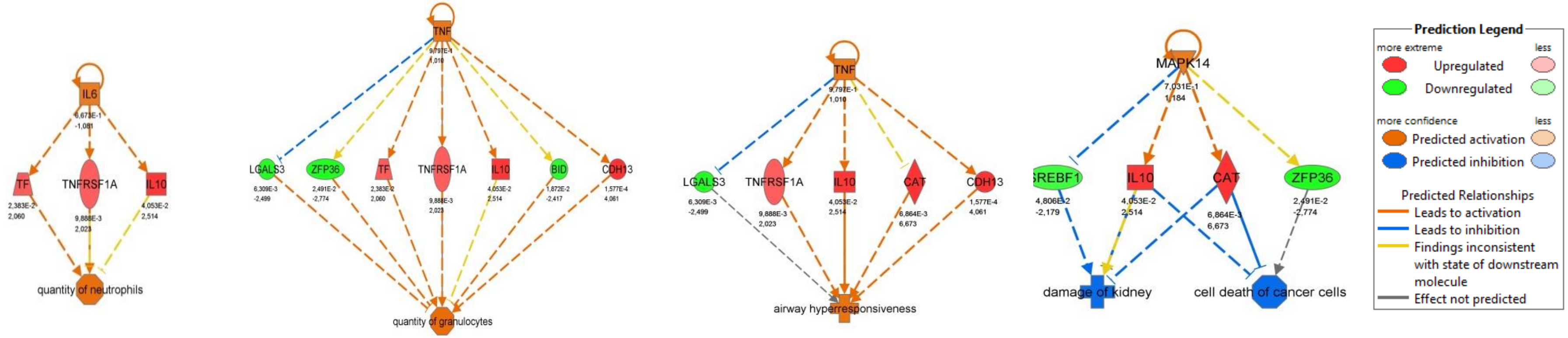

Supplemental Figure 4. Comparative Analysis of downstream processes up- or downregulated in BPD depending on disease severity (Ingenuity Pathway analysis)

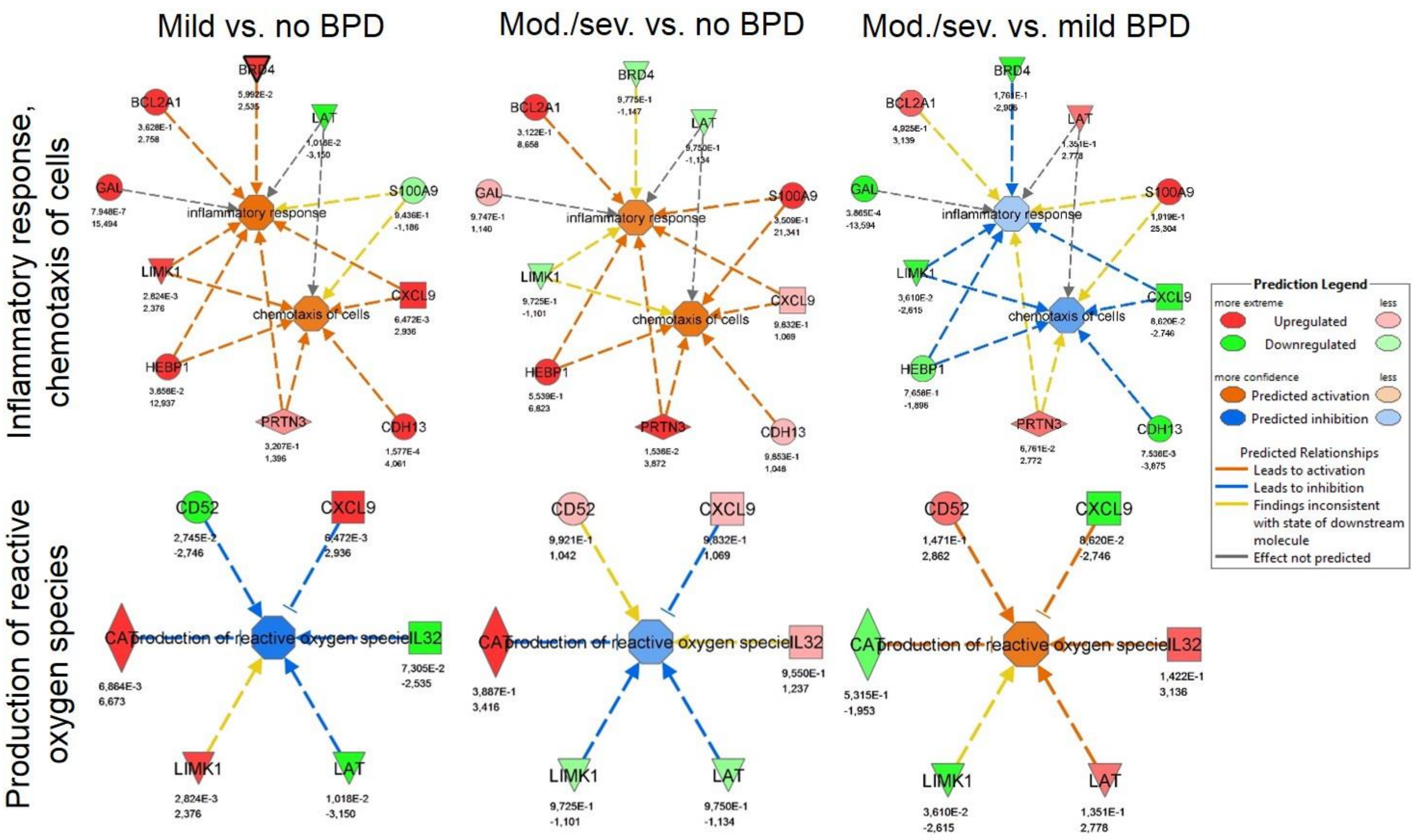

Supplemental Figure 5. Protein concentration in umbilical blood of preterm infants with BPD (n=45) and BPD (n=55) (21-plex premixed human cytokine milliplex panel (HPANLXM 2, Luminex® xMAP®, Luminex, TX, US).

Data was transformed using the logarithm to the base of 10, outlier values represented as dots (any values over 1.5 times the interquartile range over the 75th percentile or any values under 1.5 times the interquartile range under the 25th percentile).

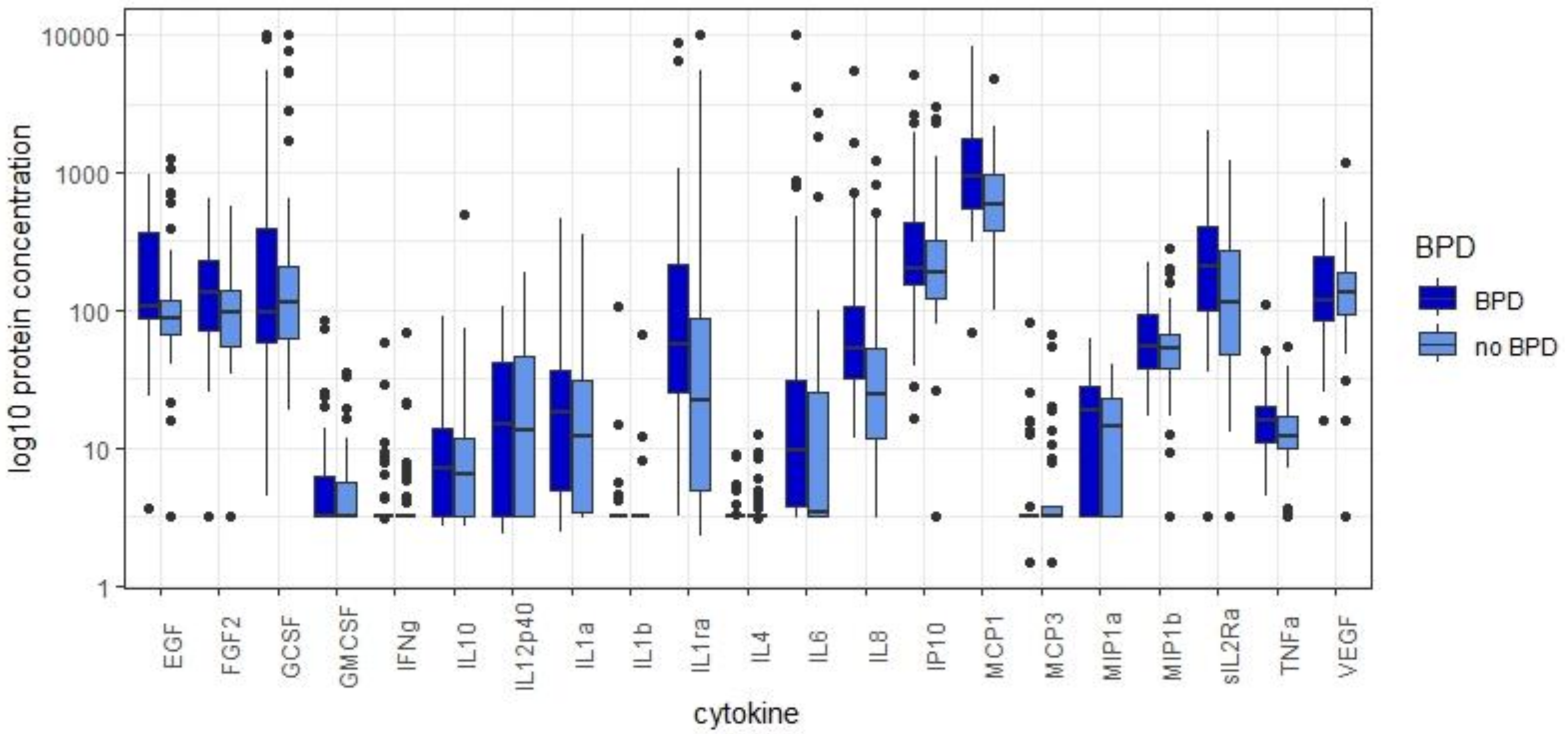

Supplemental figure 6: Gating strategy example of monocyte subgroups.

Monocyte subgroups were identified by gating on CD14/HLA-DR positive cells (C) out of all CD45 positive leukocytes (A, B). Further analysis for the expression of CD14 vs CD16 allowed us to define classical monocytes with high CD14 expression (CD14++CD16+), non-classical monocytes with lower CD14 and high CD16 expression (CD14+CD16+) and intermediate monocytes with high CD14 and lower CD16 expression (CD14++CD16+). Fluorescence-labeled counting beads enabled the calculation of absolute cell counts per µl whole blood (B). The example provided here show cased the results obtained in a sample of a preterm infant (GA 26.4 weeks) with severe BPD on day of life 13 (during ventilation and oxygen supply). Monocyte subgroups classified as classical monocytes with 1013/µl whole blood (38% of all monocytes), non-classical monocytes with 82/µl (3.1% of all monocytes) and intermediate monocytes with 1560/µl (58.7% of all monocytes)(D). The corresponding isotype control IgG1-PE for CD16 demonstrated the specificity of the CD16 staining (E).

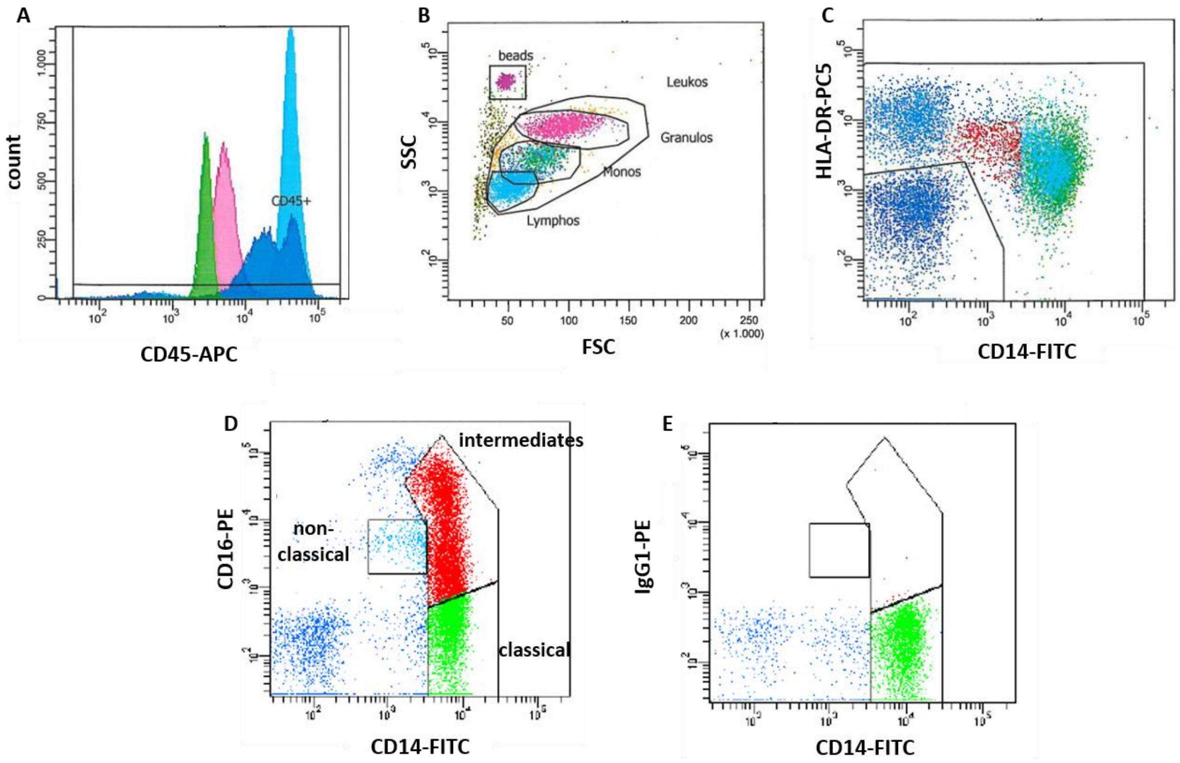

Supplement: Supplementary file 1 [file DataSheet_1.pdf]
